# Supplementary figures and images for: A reaction-diffusion network model predicts a dual role of Cactus/IκB to regulate Dorsal/NFκB nuclear translocation in Drosophila
Source: PLoS Comput Biol. 2021 May 27;17(5):e1009040. doi: 10.1371/journal.pcbi.1009040 (PMC8189453; doi:10.1371/journal.pcbi.1009040)

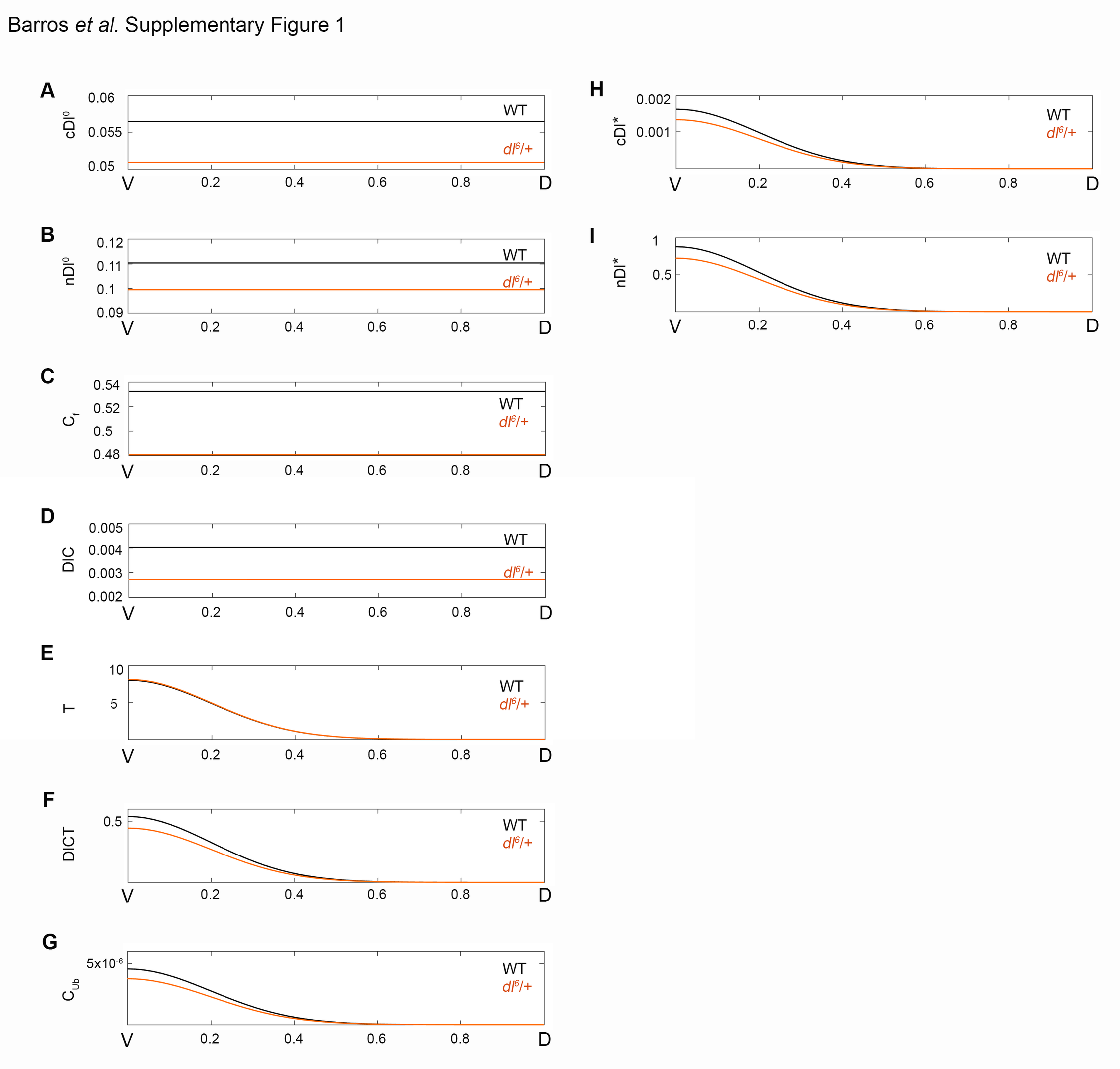

Supplement: S1 Fig — Distribution of free cytoplasmic (cDl0, A) and nuclear (nDl0, B) Dorsal, free Cactus (Cf, C), DlC complexes formed by Dl dimer and Cact monomer (D), Toll (T) receptor (E), DlCT complexes including DlC and a Toll receptor (F), Cactus (Cub, G) and cytoplasmic Dorsal (cDl*, H) modified by Toll Pathway (G-H), nDl modified by Toll dependent pathway (I). (TIF) [file pcbi.1009040.s001.tif]

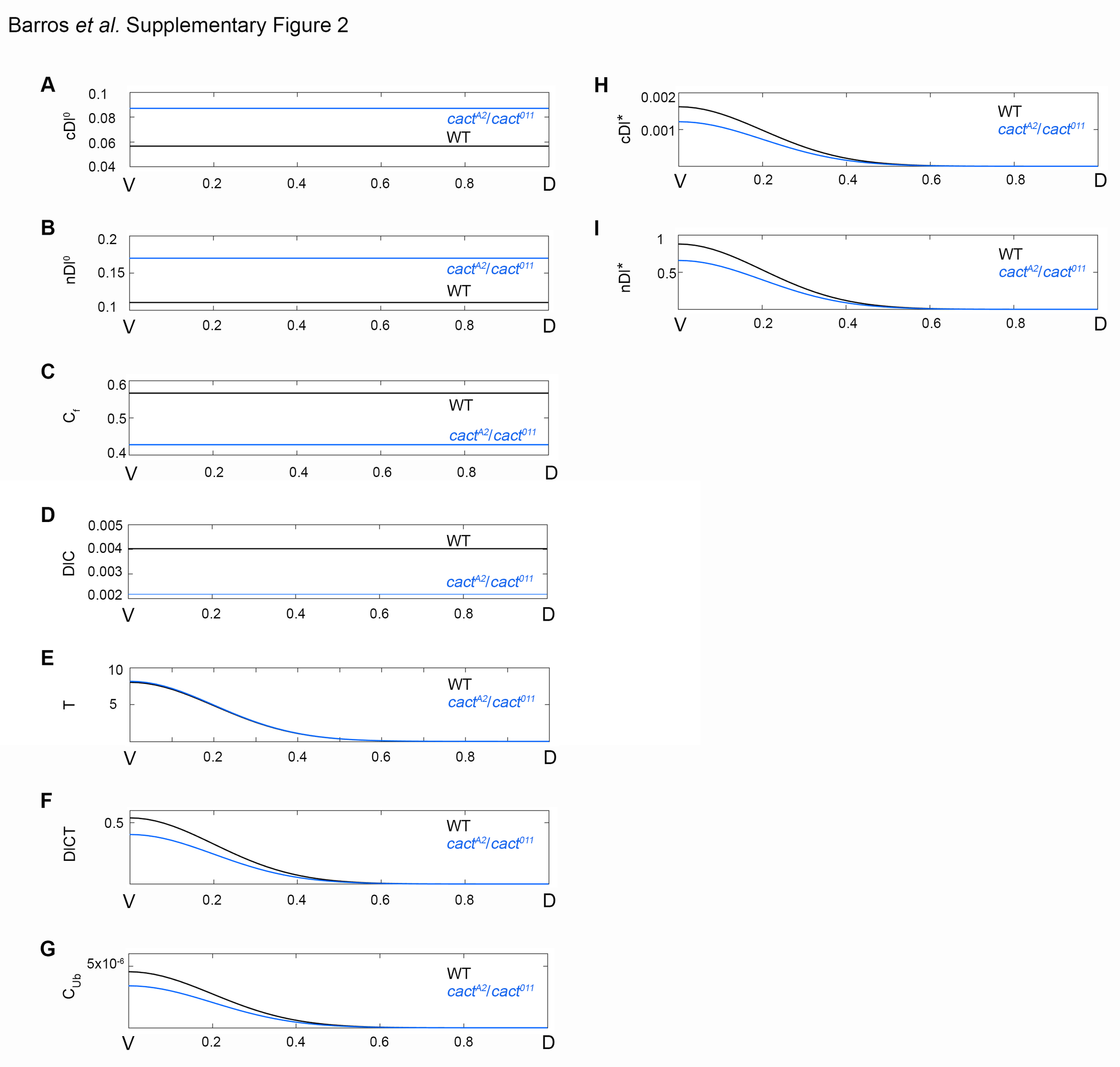

Supplement: S2 Fig — Distribution of free cytoplasmic (cDl0, A) and nuclear (nDl0, B) Dorsal, free Cactus (Cf, C), DlC complexes formed by Dl dimer and Cact monomer (D), Activated Toll (T) receptor (E), DlCT complexes including DlC and an activated Toll receptor (F), Cactus (Cub, G) and cytoplasmic Dorsal (cDl*, H) modified by Toll Pathway (G-H), nDl modified by Toll induced (I). (TIF) [file pcbi.1009040.s002.tif]

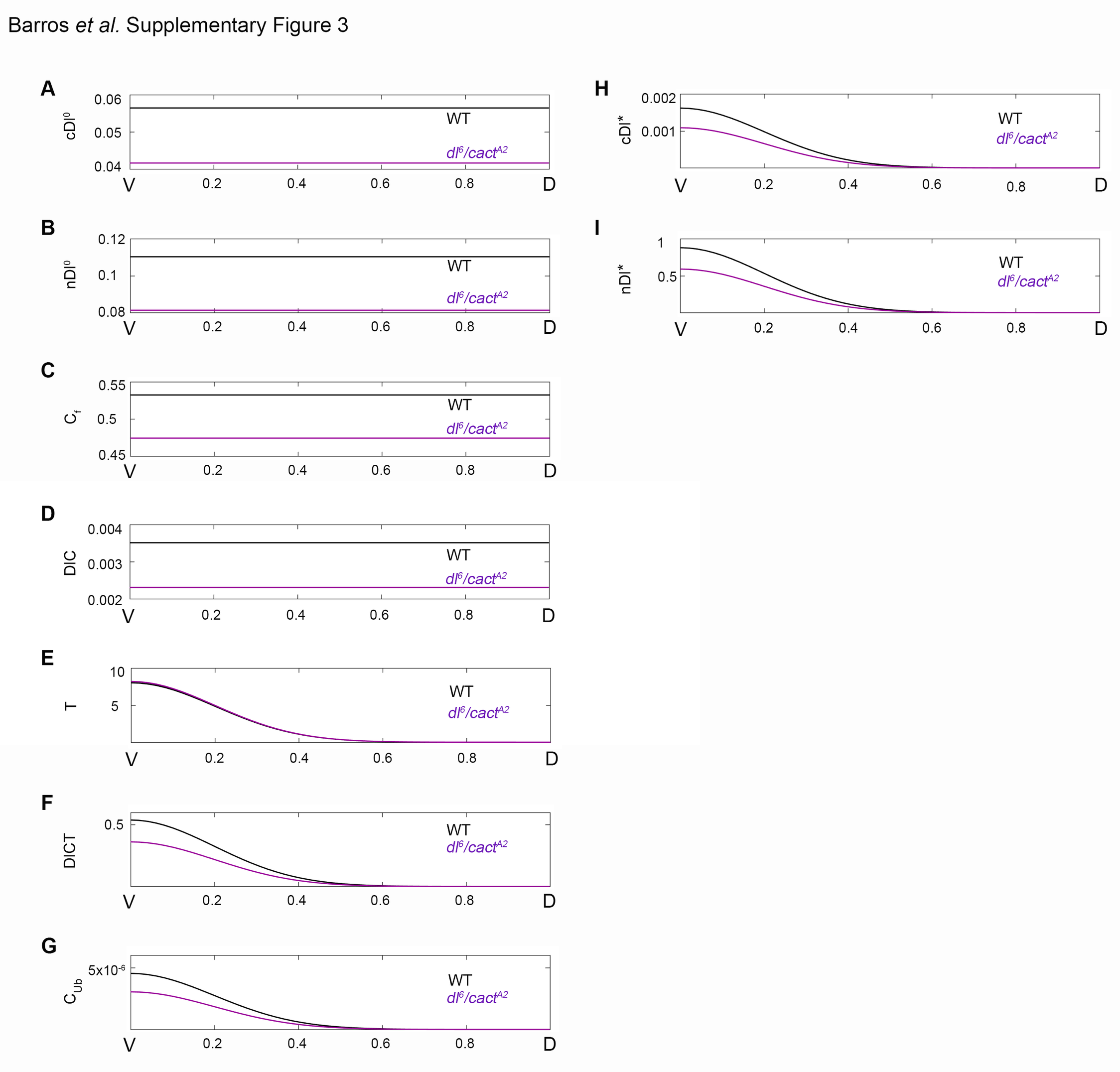

Supplement: S3 Fig — Distribution of free cytoplasmic (cDl0, A) and nuclear (nDl0, B) Dorsal, free Cactus (Cf, C), DlC complexes formed by Dl dimer and Cact monomer (D), activated Toll (T) receptor (E), DlCT complexes including DlC and an activated Toll receptor (F), Cactus (Cub, G) and cytoplasmic Dorsal (cDl*, H) modified by Toll Pathway (G-H), nDl modified by Toll induced (I). (TIF) [file pcbi.1009040.s003.tif]

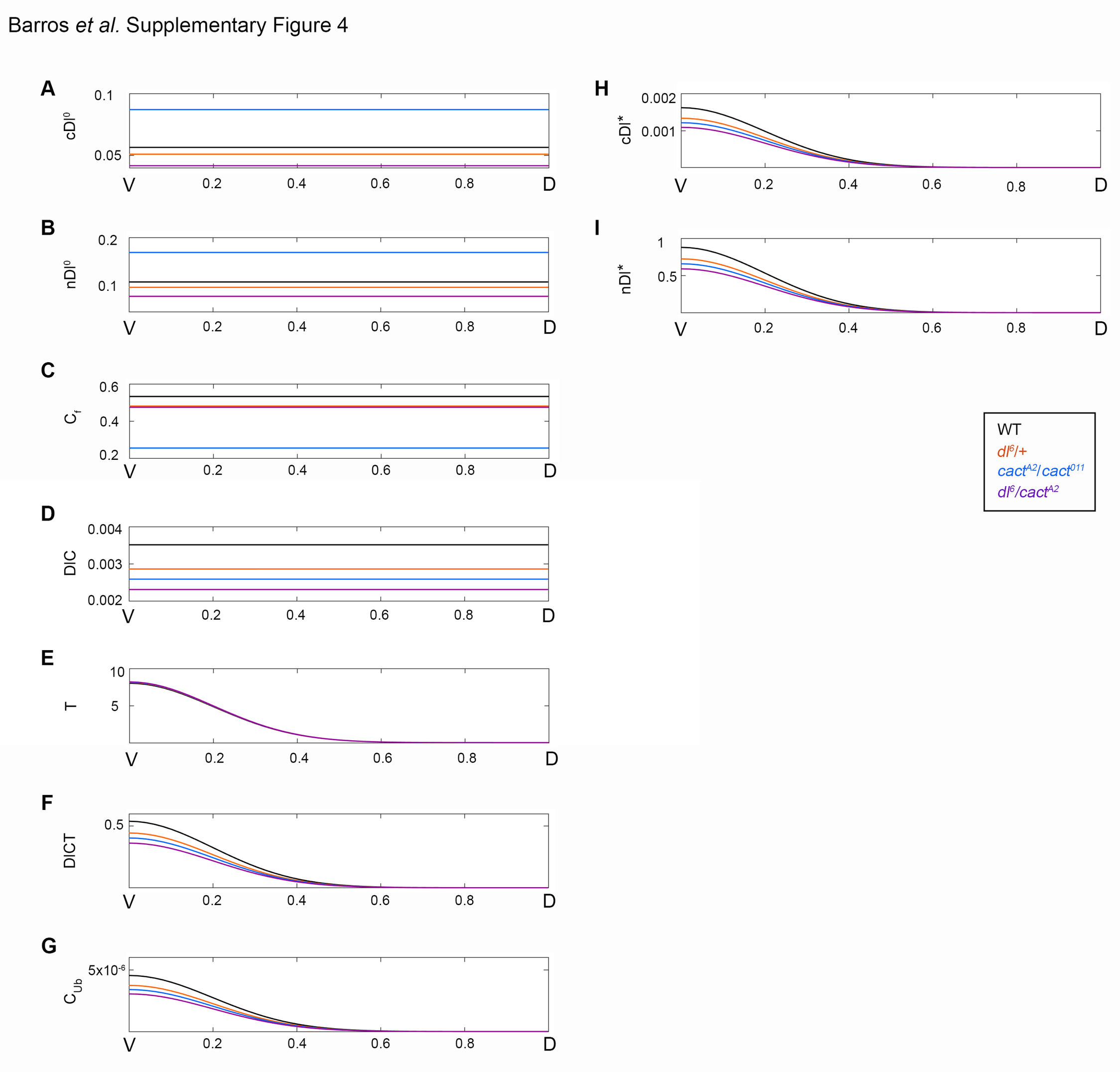

Supplement: S4 Fig — Curves for wild-type (black), dl6/+ (orange), cactA2/cact011 (blue), and dl6/cactA2 (purple) mutants. Distribution of free cytoplasmic (cDl0, A) and nuclear (nDl0, B) Dorsal, free Cactus (Cf, C), DlC complexes formed by Dl dimer and Cact monomer (D), activated Toll (T) receptor (E), DlCT complexes including DlC and an activated Toll receptor (F), Cactus (Cub, G) and cytoplasmic Dorsal (cDl*, H) modified by Toll Pathway (G-H), nDl modified by Toll induced (I). (TIF) [file pcbi.1009040.s004.tif]

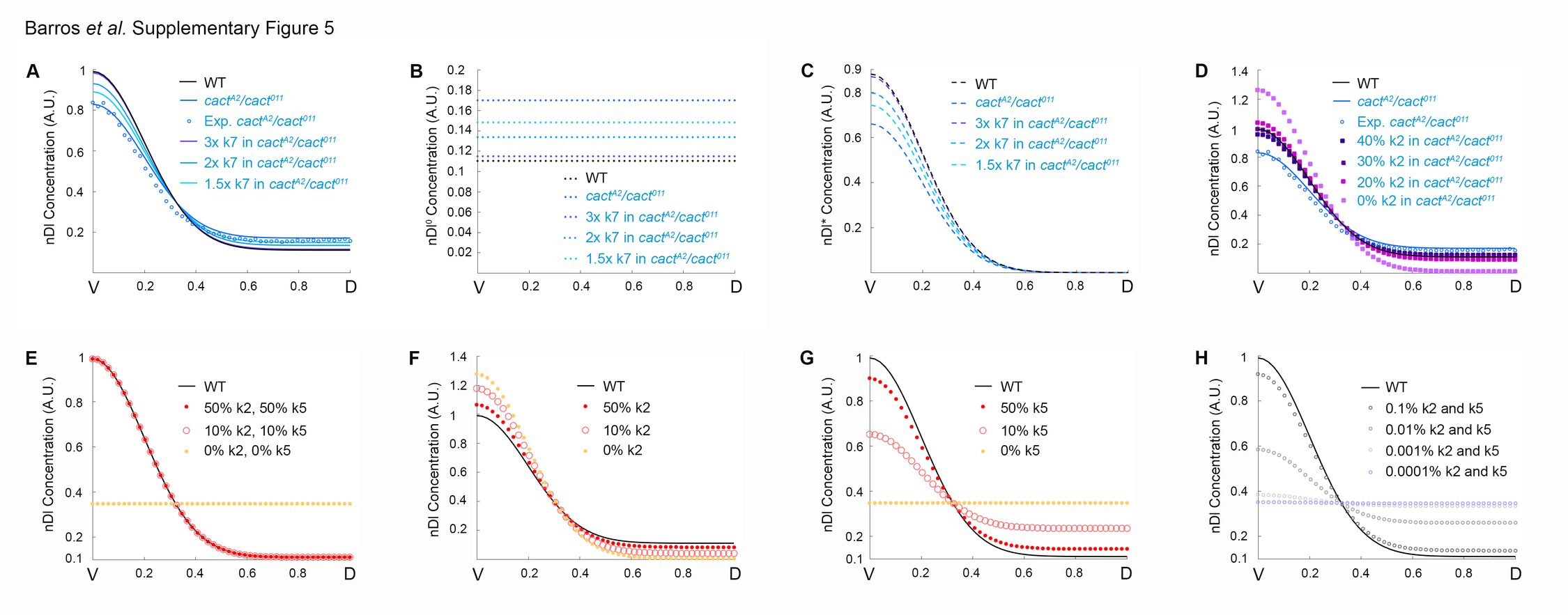

Supplement: S5 Fig — (A-C) Model prediction of nuclear species distribution for 3x, 2x or 1.5x increases of kinetic constant k7 in a cactA2/cact011 mutant background. nDl, total nuclear Dorsal dimers (A); nDl0, free nuclear Dorsal dimers (B); nDl*, nuclear Dorsal dimers induced by activated Toll (C). (D) nDl simulation decreasing k2 by 20%, 30%, 40% or nulling k2 (0%) in a cactA2/cact011 mutant background comparing to experimental data (circle symbol) and WT and cactA2/cact011 mutant simulations. Simultaneous reduction of k2 and k5 by 0%, 10% and 50% (E) or by 0.1%, 0.01%, 0.001% and 0.0001% (H) comparing to control (WT) nDl concentration simulations (E, H). Reduction of k2 (F) or k5 (G) by 0%, 10% and 50%. (TIF) [file pcbi.1009040.s005.tif]

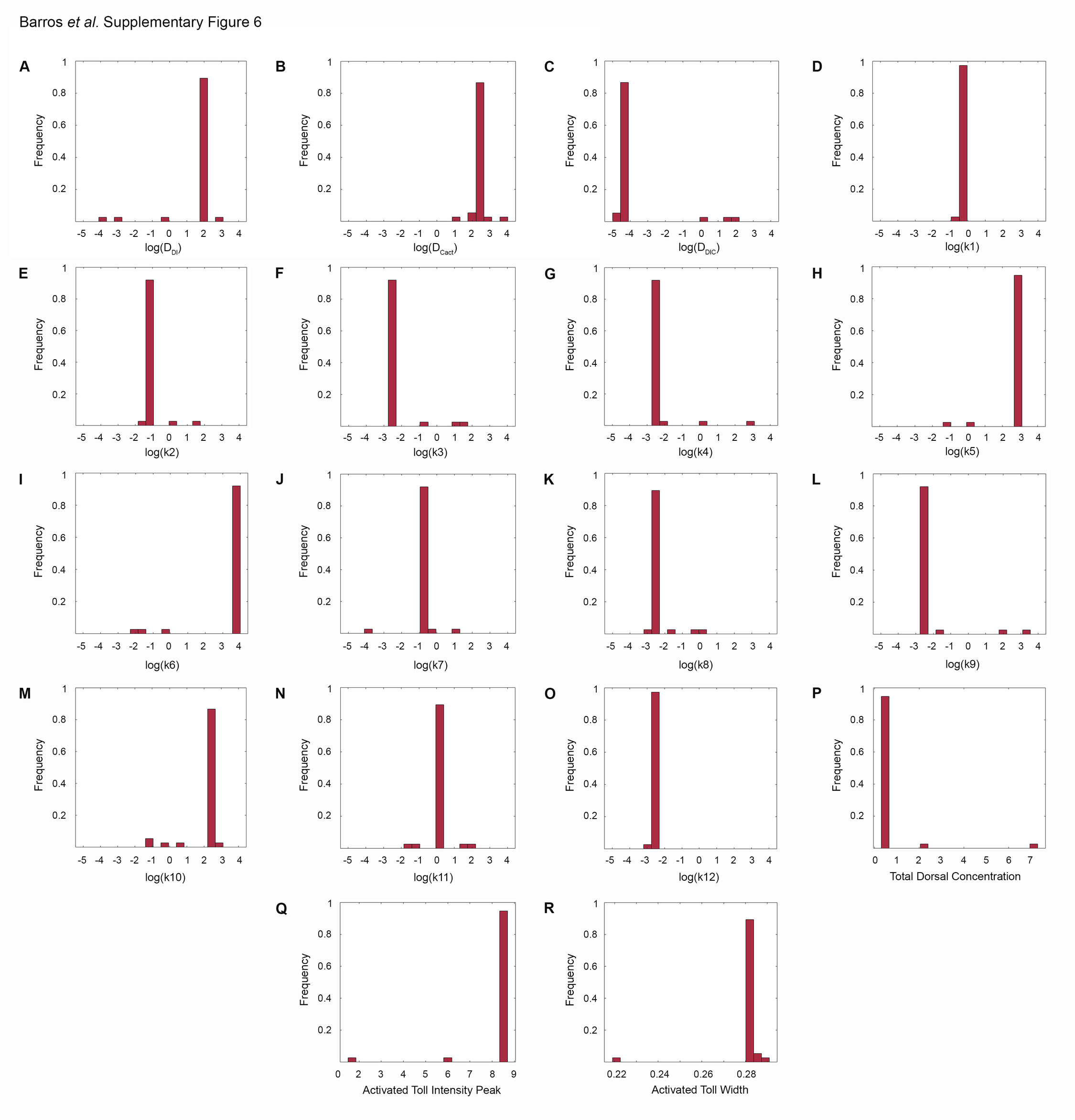

Supplement: S6 Fig — We selected the 37 best individuals from the full Genetic Algorithm execution, following the criterion of the cost function being less than or equal to 0.069. (A-C) Diffusion coefficients, in logarithmic scale; (D-O) kinetic constants, in logarithmic scale; (P) peak activated Toll concentration; (Q) activated Toll width; (R) total Dorsal concentration. (TIF) [file pcbi.1009040.s006.tif]

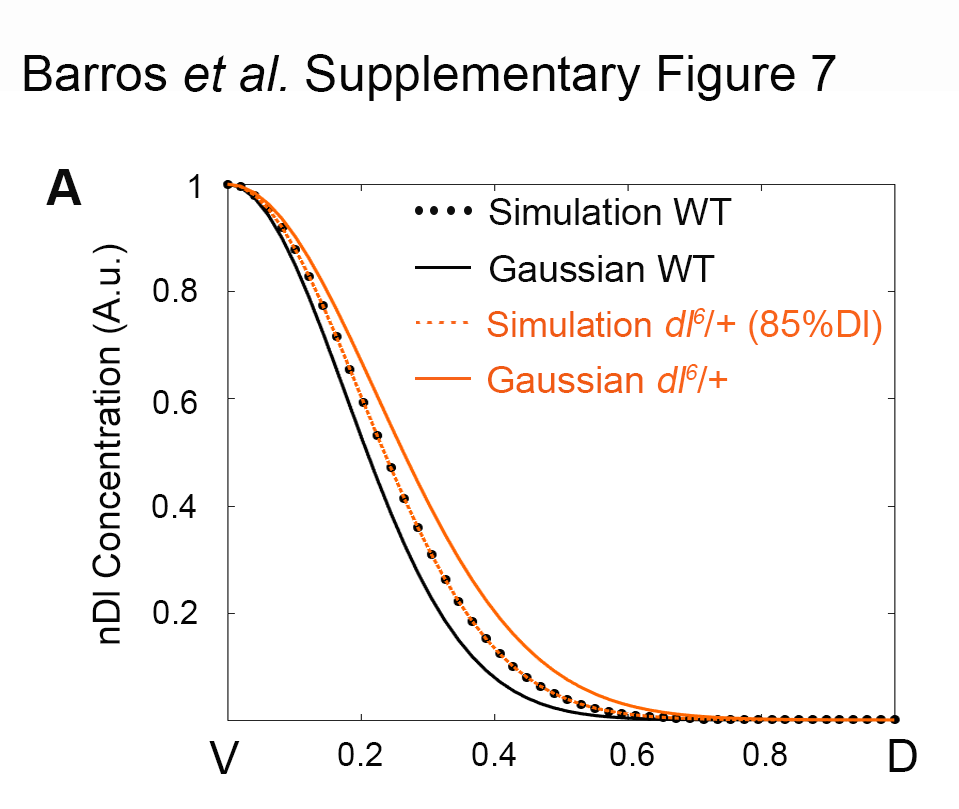

Supplement: S7 Fig — Model concentration distribution of nuclear Dl (dotted lines) was normalized in order to compare the shape of the profiles obtained for the wild-type and dl6/+ genotypes. As a reference, Gaussian curves were fitted to the experimental data (solid lines). (TIF) [file pcbi.1009040.s007.tif]
