# Supplementary material for: A reaction-diffusion network model predicts a dual role of Cactus/IκB to regulate Dorsal/NFκB nuclear translocation in Drosophila
Source: PLoS Comput Biol. 2021 May 27;17(5):e1009040. doi: 10.1371/journal.pcbi.1009040 (PMC8189453; doi:10.1371/journal.pcbi.1009040)
Supplement: Attachment — Submitted filename: Answers to reviewers.pdf [file pcbi.1009040.s010.pdf]

Answers to the reviewer's questions/comments.

Dear PLoS Computational Biology editors,

We would like to thank all reviewers' comments. We have prepared a revised manuscript, which we hereby submit. Here, we answer in detail all questions/comments raised by the reviewers.

We submitted a *Revised manuscript (marked-up copy)* showing all changes we have made to the original version of the manuscript. This file was submitted as a "*Related Manuscript*" file, as required by the submission guidelines. Also, we submitted a clean copy of our revised manuscript that does not show our changes. We upload this as the "*Manuscript*" file, as required by the submission guidelines.

Cordially,

Francisco J. P. Lopes
Associate Professor
Interdisciplinary Research Centre, UFRJ Caxias
flopes@ufrj.br

Reviewer #1:
This paper refines previous mathematical models of Dorsal/NFkB gradient formation in Drosophila. The authors claim that their new model predicts a dual role of Cactus/IkB in inhibiting and promoting Dorsal nuclear translocation. The model is inspired by experimental observation with certain hypomorphic cactus allele combinations (Cardoso et al., 2017).

> The reviewer is correct; we have generated a new mathematical model that includes a new mechanism not yet described in previous theoretical models. This new mechanism is based on experimental data from our Cardoso et al paper and many other data previously published by others. Importantly, in Cardoso et al, we analyzed several loss-of-function combinations (Suppl.

Table 1), including alleles and deficiencies studied in Roth et al, 1991, 1995 and Bergman 1996) and found that some of the data in Cardoso et al seemed to conflict with previously published data. In fact, it was the subject of extensive discussion with Development journal referees. We kindly ask Reviewer #1 to go back to the Cardoso et al paper, where the results of this discussion are presented in detail. Importantly, we were able to show that apparent discrepancies between ours and earlier results resides in the modern quantitative technologies we used, and that were able to reveal a novel function for Cactus.

New mechanisms included in the proposed model are the Dorsal-dependent translational control of Cactus (I could not trace the experimental evidence for this),

> The Dorsal-dependent translational control of Cactus was shown by Kubota and Gay in 1995, which is referred to in the text and is part of the bibliography provided. In fact, this paper was already cited in the previous version of the manuscript, page 5, after " We take into account non-uniform activation of Toll as in current models, two Dl nuclear translocation mechanisms, translational regulation of Cact by Dl [29, 30]", 30 being Kubota and Gay 1995.

the processing of Cactus (probably CalpA-dependent) and a Toll-dependent mechanism by which Cactus promotes Dorsal nuclear transport.

> A clarification is critically needed here. We actually suggested the inverse, that a Toll Independent mechanism of Cactus regulation controls Dorsal nuclear import, which was the main result of Cardoso et al. In our present paper, we used a new theoretical model to propose a molecular mechanism for this novel result. In fact, we believe that we made clear in the manuscript that a Toll-independent mechanism is being described. See, for example, the following sentence from Reviewer #2 "… This Toll-independent Cact regulation was crucial to elucidate the dual role of Cact – prevent and promote translocation of Dl protein."

The model has 18 parameters (12 kinetic constants, 3 diffusion coefficients, the total Dl concentrations and 2 activated-Toll related parameters). These are fitted to reproduce the experimentally measured Dorsal nuclear distribution using a genetic algorithm. Does this procedure always lead to the same stable outcome? How much parameter variation did the authors observe upon repeated runs of the optimization process?

>We greatly appreciated this question, as we realized that a discussion about this issue was missing in our manuscript. We tried to address this issue to the best of our knowledge by improving the Method's sub-section "Model calibration and simulation" lines 699 to 721 and including the new Supplementary Fig. 6.. We kindly ask for the referee to check the new section included.

My main criticism however concerns the fact that the authors did not correctly incorporate published literature.

> Below we discuss some specifics related to this criticism. It is important to point out that the early analysis that revealed how the Dl gradient is established in response to Toll pathway activation relied on cuticle pattern and non-quantitative analysis of the Dl gradient. Using a quantitative approach, we were able to reveal aspects of the Dl gradient that had remained hidden in previous works due to technical restrictions. For this reason, some of the results in Cardoso 2017 came as a surprise to researchers that followed or were part of these early endeavors.

We do agree with the reviewer strategy to compare our results from Cardoso 2017 with the early work performed by many labs that defined how the Dorsal gradient is established in the

Drosophila embryo. We are aware of all the allelic combinations studied then, and their effects on the embryonic cuticle, the nuclear Dorsal gradient, and Dl-target genes. However, we must point out that, by performing a quantitative analysis of the Dorsal gradient, we observed patterns that had not been detected. We clearly observed a decrease in ventral nuclear Dorsal in cact loss-of-function, as revealed in the graphs in Figure 1, Suppl Fig 1 and Suppl Fig 2 of Cardoso et al, where we thoroughly analyzed several cact allelic combinations. The differences in ventral levels of nuclear Dl that we showed could not be perceived in the early works of Roth 1989, 1991, Belvin, 1995 or Bergman 1996, since Dl gradients were revealed with immuno-histochemical methods, as compared to quantitative fluorescent antibody detection and in situ hybridization.
Therefore, we, and the reviewers of Cardoso 2017, are convinced of the effects of cact loss-of-function in decreasing ventral nuclear Dl levels and consequent decrease in the size of the domain of ventral genes such as snail and twist.

Their crucial simulation of the cactus null condition (Figure 4 E) leads to a uniform nuclear Dorsal distribution (corresponding to dorsolateral DV positional values!). However, this cannot be the case. They cite the paper by Bergmann et al., 1996 in which independent germ line clone experiments with three amorphic loss of function alleles of cactus were described. One of them is a defined genomic deletion of the entire cactus gene. In all three cases Bergmann et al observed nuclear Dorsal gradients, albeit with a decreased slope Thus, polarity was not lost.
Most importantly there was no indication of decreased ventral nuclear Dorsal concentrations compared to wildtype.

> The most important data in Bergmann et al., 1996 to support the referee's argument is shown in Fig. 8, the sole figure where the Dl gradient in a cact loss-of-function mutant is shown. The experiment that produced these results has two important characteristics that greatly differentiates from the experiments used in our paper:
1. They used immuno-histochemical methods
2. There was no quantitative analysis of the Dorsal gradient.

Since this may preclude detection of differences in high nDl amounts in ventral regions of the embryos, it makes no sense that Fig. 8 experiment (in Bergmann et al 1996) could be used to challenge fluorescent immunostaining assays combined with rigorous computational image analysis of Cardoso et al, 2017, as detailed below. All other data cited in the Bergman 1996 paper using germline clones for loss-of-function cact alleles is either not shown or is presented as embryonic cuticles, thus no further information on the Dorsal gradient is provided that could enable comparison with data shown in Cardoso 2017.

Bergman 1996 generates germline clones for three alleles:
- cact[255], a P-element insertion and likely hypomorph, they cite observing a V1 cuticle phenotype but no figure is shown;
- cact[III18] is Df(2L)III18, which deletes cact, fzy and chi genes, plus recoded fzy and chi. They show a shallow Dl gradient for these germline clones and cite a V1 cuticle phenotype;
- cact[D13], that has a premature stop codon, they cite a V1 cuticle phenotype but no figure is shown.

Based on cact[III18] germline clones they observe an expansion of nuclear Dl in dorsal positions of the embryo, in agreement with our own loss-of-function analysis, where Dl is expanded dorsally, as well as expression of the lateral target gene sog. On the other hand, the Dl gradient shown for the cact[III18] germline clone does not allow to evaluate the Dl gradient in ventral regions of the embryo, a limitation of the staining technique, as it does not allow the linear assessment and thus quantification of the signal. Besides that, our quantitative analysis using fluorescent detection shows that loss of cactus function in the strong loss-of-function allele cact[A2] over a deficiency for cact results in ventral loss of peak nuclear Dorsal levels.

>However, we agree with the reviewer that our results, as well as those in the literature, do not show complete loss of polarity in cactus mutants. For that reason, we are grateful to the reviewer

in pointing out an inconsistency in our writing. Fig. 4E shows model simulations for different levels of Cact reduction, from 80 to 100% reduction. All those simulations agree with our experimental data (Cardoso 2017) except the limit situation where Cact is completely absent. This discordance clearly indicates that there are additional mechanisms that may still need to be included in the model. However, as with any model, we understand that our model is limited in the sense that it is not a perfect reproduction of the biological system.

We also realize that our writing may have generated this misunderstanding, therefore we have modified the sentence referring to Fig 4E where it now reads:
"....the model correctly predicts that loss of cactus function progressively leads to flattening of the Dorsal gradient, reflecting the pattern of embryos with expanded lateral territories"(Cardoso, 2017)

Furthermore, to make it clear to the reader, and based on the results of cact germline clones, as pointed out by the reviewer, we have added the sentence:
"On the other hand, the complete absence of Cact (0%) leading to apolar embryos has no support in the literature. Germline clones for loss-of-function cact alleles, which lead to the highest degree possible of cactus loss, still retain some polarity, suggesting that other processes are involved in gradient formation, such as Dorsal phosphorylation (Govind et al, 1993), not reflected in our model."

Early germline clones by pole cell transplantations using one of these amorphic alleles (a cactus point mutation with an early stop codon deleting all ankyrin repeats) led to embryos with slightly expanded twist domains (Roth et al. 1991).

> the fact that cactus performs an inhibitory role, as evident in the experiment with the cact[D13] allele cited above by the reviewer, is clear in the literature and also in our own papers. This does not exclude, however, that cactus also displays an additional, positive role. This is extensively discussed in Cardoso et al, 2017 and also in Fontenele et al, 2013, and should take into account the ***relative levels*** *of Cactus to Dorsal.*

Further fundamental mistakes of the current paper are the comparative simulations of pelle and Toll KD embryos. The experimentally observed null mutations of these genes lead to identical phenotypes with embryos lacking nuclear Dorsal to the same degree (Roth et al., 1989). It seems that the authors assume that Tollrm9 is a null allele, which in fact it is lateralized Toll hypomorph.

> Thanks to the reviewer's comments we realize that this section of the manuscript was unclear. While this section is not the central issue in our manuscript, we are at fault for not having stated our reasoning behind the simulations in Fig 7. We wanted to simulate two different conditions that impair Toll signals: the first condition blocks transduction of the Toll signal at the level of the kinase that leads to Cactus phosphorylation, ubiquitination and consequent degradation; the second condition impairs Toll activation that is required for mobilizing Toll-responsive signaling complexes. The first condition mimicked well a pelle loss-of-function mutant, which generates dorsalized embryos, with a phenotype similar to a Toll loss-of-function. It is important to note that dorsalizing Toll phenotypes either result from null alleles or from recessive alleles that display mutations in the intracellular domain, probably impairing signal transduction.

The second condition blocks Toll signals at the receptor level, by impairing Toll activation that is necessary to mobilize signaling complexes, prior to transduction of the Toll signal. Surprisingly, this condition resulted in a nDl phenotype that recalls the phenotypes of lateralizing Toll alleles Tl[rm9] and Tl[rm10], which are alleles that carry nucleotide substitutions in the region encoding the Toll receptor extracellular domain (Schneider at al, 1991). Such mutations in the Leucine-rich domains probably impair the interaction between homodimers and decrease the mobilization of signaling complexes. Although we were unable to reproduce the Toll null phenotype by decreasing the level of "activated Toll", exploring the lateralized phenotype that resulted may give us insight into the most important parameters that vary in this condition.

To make this section clear, as well as the respective figure legends, we have modified the text accordingly (Figure 7 legend and first paragraph at page 19). We thank the reviewer for pointing this out.

In their simulation the null mutants of Cactus and that of Toll lead to almost identical phenotypes which both do not agree with experimental observations. The fact that their simulations lead to results agreeing with the wrong phenotypes, casts serious doubt on the entire approach.

> As discussed above, we are not exploring in Fig 7 the phenotype of a Toll null, but rather a Toll condition that leads to lateralized embryos, and that helps understand what are the fundamental parameter changes that lead to expansion of lateral gene expression territories, as seen in cact allelic combinations and in Tl[rm9].

All available evidence suggests that Dorsal protein (monomer or dimer) can be subject to Toll induced phosphorylation in absence of Cactus and that this process is required to achieve the highest nuclear Dorsal concentrations. This explains why cactA2; Toll double mutants are lateralized at a ventrolateral level, while cactA2; Tollrm9 is mesodermalized (uniform highest nuclear Dorsal) (Roth et al., 1989).

> Indeed, the phosphorylation of Dl protein has an important role in the Toll pathway and is an additional aspect that would be very interesting to include in further models. However, in this manuscript we have concentrated in modeling the effects of Cactus, particularly considering the novel effects we have observed from our quantitative analysis of several cact mutant combinations.

Reviewer #2: Barros et al used a modeling approach to characterize the kinetics of nuclear Dorsal (Dl) gradient in early Drosophila embryos, and showed that the Dorsal-Cactus complex formation independent of Toll-signaling pathway is a key step to be added in the model to fully reproduce experimental data on nuclear Dl expression. Typically, it is known that Cactus (Cact) binds to Dl and blocks Dl's entry into the nucleus. Upon activation of Toll signaling pathway, Cact is phosphorylated and degraded, allowing freed Dl to enter the nucleus. The authors had recently shown that Cactus actually has an additional role to promote Dl nuclear translocation, especially in the ventral region where Toll signal is high. Since the mechanism of Cact's dual role in nuclear Dl regulation is not known, they used a new reaction-diffusion based model to characterize Dl dynamics. Using the model, they were able to show that in addition to Toll-dependent Dl translocation, a fraction of Dl-Cact complexes work independently of Toll pathway, and unbinding of Cact from the complex would allow direct entry of Dl into nucleus. This Toll-independent Cact regulation was crucial to elucidate the dual role of Cact – prevent and promote translocation of Dl protein. In cact mutant embryos, reduced Cact level would form fewer Dl-Cact complex, and consequently reduced number of Dl molecules can be freed from the Dl-Cact complex by activated Toll pathway, resulting in lower Dl level in cact mutant embryos, especially in ventral region. In the lateral/dorsal region where Toll signal is low, however, reduced Cact would allow more free Dl to directly enter nucleus, increasing basal Dl level in cact mutant embryos (dominant Toll-independent Cact regulation). Their model agrees with experimental data, including WT and various dl and cact mutant conditions. The manuscript is written logically, and dl/+, cact/cact, and dl/cact mutant data were used appropriately to test dual role of Cact and to better understand underlying mechanism of Cact regulation.

Yet, there are some comments and questions I have on the manuscript that could improve this manuscript's quality and clarity.

1) In Fig 7C, and SF1, how significant are these changes in basal levels of nDl or Cactus? For example, is the difference between 0.002 vs 0.003 significant (the authors claim that in some

figure panels)? Does the trend hold with respect to some changes in parameters, or is this very sensitive to a certain set of parameters only?

Unlike 7C, the variations in 7F are significant as DlC ranges from 0.004 to 0.032. However, in both cases these variations contribute to significant changes in the levels of nDl (Fig. 7A, D) and in the levels of DlCT (Fig. 7B, E). In regard to parameter sensitivity, we conducted a detailed study as explained below.

2) Regarding the first question, how robust is the parameters with respect to changes?

> To answer to this question and improve the manuscript in respect to parameter sensitivity, we included four new paragraphs starting at page 30, line 698. The histograms show that most of the best individuals found by the Genetic Algorithm were very similar in the parameter space, indicating considerable stability of the solution, even when using an epidemic strategy to prevent the population from being dominated by solutions within a local minimum

3) Based on which data did authors use lower Cactus concentration to simulate dl6/+ condition in the model? I understand lower Dl level, but I missed the evidence for lower Cactus level in dl mutant

> The reduction in Cactus concentration in the model as a consequence of reducing Dl levels is considered in the first reaction $cDl^0 \rightarrow cDl^0 + C_f$. *We introduced this reaction based on several papers that report the decrease in Cactus levels in response to a decrease in Dorsal levels, seen both in western blots and antibody staining in whole mount or transversely cut embryos (Kubota and Gay, 1995; Belvin, 1995; Bergman, et al 1996). Importantly, Kubota and Gay, 1995 suggested that Dorsal regulates either cact mRNA stability or translation. This paper was cited in the previous version of the manuscript, page 5, after " We take into account non-uniform activation of Toll as in current models, two Dl nuclear translocation mechanisms, translational regulation of Cact by Dl [29, 30]", 30 being Kubota and Gay 1995.*

4) In Fig 3D, why does nDl level lower in lateral/dorsal side as well (it wasn't so in dl6/+)? Is there any experimental data to support the model data?

> Our dl[6]/+ experimental results, where Dl levels are decreased to 85% of wild type levels, show no decrease in nDl levels dorsally. It is possible that this difference between experimental results and the simulation shown in Fig 3D reflects a more significant decrease in total Dorsal protein than that attained in dl[6]/+. Accordingly, quantification experiments using a different dl loss-of-function allele (dl[1]) (Lieberman et al 2009) have shown a reduction in nDl levels on the dorsal side.

5) In the author's previous paper (2017 Development), the authors observed lateral expansion of Dl in cact mutant embryos. But, I don't see such evidence in the new experimental data in Fig 4B. Any comment on that?

>In Fig 4B, and 4A we show the gradient of cact[A2]/cact[011] embryos, which result in a more subtle increase in lateral nuclear Dorsal levels, compared to cact[A2]/Df. These two genotypes are explored in Cardoso et al Figs 1C and Fig 1D, respectively. Importantly, both allelic combinations lead to an expansion of lateral territories, as shown in Cardoso 2017, Fig 1K and 1H. Since the deficiency (Df) used in cact[A2]/Df also deletes loci that are not included in our model we have chosen not to simulate this condition in the current manuscript.

6) It took a while to understand how DlCT and DlC explains dual role of Cact. It would be nice if the authors clarify it a bit more. Personally, I understood it only after reading Discussion section. Maybe use some of these phrases in Results, when they first talked about it?

> *We have added a few sentences explaining this dual role of Cactus in the results section, as suggested by the reviewer (page 14, end of first paragraph, line 295 to 299).*
