# Supplementary material for: A reaction-diffusion network model predicts a dual role of Cactus/IκB to regulate Dorsal/NFκB nuclear translocation in Drosophila
Source: PLoS Comput Biol. 2021 May 27;17(5):e1009040. doi: 10.1371/journal.pcbi.1009040 (PMC8189453; doi:10.1371/journal.pcbi.1009040)
Supplement: Attachment — Submitted filename: Answers to all reviewers.pdf [file pcbi.1009040.s011.pdf]

Answers to the reviewer's questions/comments.

Rio de Janeiro, December 22th, 2020

Dear PLoS Computational Biology editors,

We would like to thank all reviewers' comments. We have prepared a revised manuscript, which we hereby submit. Here, we answer in detail all questions/comments raised by the reviewers.

We submitted a *Revised manuscript (marked-up copy)* showing all changes we have made to the original version of the manuscript. In this file, blue color revisions are related to the comments by reviewers #1 and #2. Red color revisions are related to comments by reviewers #3 (second round of revision). This file was submitted as a "*Related Manuscript*" file, as required by the submission guidelines. Also, we submitted a clean copy of our revised manuscript that does not show our changes. We upload this as the "*Manuscript*" file, as required by the submission guidelines.

Cordially,

Francisco J. P. Lopes
Associate Professor
Interdisciplinary Research Centre, UFRJ Caxias
flopes@ufrj.br

Reviewer #1:
This paper refines previous mathematical models of Dorsal/NFkB gradient formation in Drosophila. The authors claim that their new model predicts a dual role of Cactus/IkB in inhibiting and promoting Dorsal nuclear translocation. The model is inspired by experimental observation with certain hypomorphic cactus allele combinations (Cardoso et al., 2017).

> The reviewer is correct; we have generated a new mathematical model that includes a new mechanism not yet described in previous theoretical models. This new mechanism is based on experimental data from our Cardoso et al paper and many other data previously published by others. Importantly, in Cardoso et al, we analyzed several loss-of-function combinations (Suppl. Table 1), including alleles and deficiencies studied in Roth et al, 1991, 1995 and Bergman 1996) and found that some of the data in Cardoso et al seemed to conflict with previously published data. In fact, it was the subject of extensive discussion with Development journal referees. We kindly ask Reviewer #1 to go back to the Cardoso et al paper, where the results of this discussion are presented in detail. Importantly, we were able to show that apparent discrepancies between ours and earlier results resides in the modern quantitative technologies we used, and that were able to reveal a novel function for Cactus.

New mechanisms included in the proposed model are the Dorsal-dependent translational control of Cactus (I could not trace the experimental evidence for this),

> The Dorsal-dependent translational control of Cactus was shown by Kubota and Gay in 1995, which is referred to in the text and is part of the bibliography provided. In fact, this paper was already cited in the previous version of the manuscript, page 5, after " We take into account non-uniform activation of Toll as in current models, two Dl nuclear translocation mechanisms, translational regulation of Cact by Dl [29, 30]", 30 being Kubota and Gay 1995.

the processing of Cactus (probably CalpA-dependent) and a Toll-dependent mechanism by which Cactus promotes Dorsal nuclear transport.

> A clarification is critically needed here. We actually suggested the inverse, that a Toll Independent mechanism of Cactus regulation controls Dorsal nuclear import, which was the main result of Cardoso et al. In our present paper, we used a new theoretical model to propose a molecular mechanism for this novel result. In fact, we believe that we made clear in the manuscript that a Toll-independent mechanism is being described. See, for example, the following sentence from Reviewer #2 "… This Toll-independent Cact regulation was crucial to elucidate the dual role of Cact – prevent and promote translocation of Dl protein."

The model has 18 parameters (12 kinetic constants, 3 diffusion coefficients, the total Dl concentrations and 2 activated-Toll related parameters). These are fitted to reproduce the experimentally measured Dorsal nuclear distribution using a genetic algorithm. Does this procedure always lead to the same stable outcome? How much parameter variation did the authors observe upon repeated runs of the optimization process?

>We greatly appreciated this question, as we realized that a discussion about this issue was missing in our manuscript. We tried to address this issue to the best of our knowledge by improving the Method's sub-section "Model calibration and simulation" lines 699 to 721 and including the new Supplementary Fig. 6.. We kindly ask for the referee to check the new section included.

My main criticism however concerns the fact that the authors did not correctly incorporate published literature.

> Below we discuss some specifics related to this criticism. It is important to point out that the early analysis that revealed how the Dl gradient is established in response to Toll pathway activation relied on cuticle pattern and non-quantitative analysis of the Dl gradient. Using a quantitative approach, we were able to reveal aspects of the Dl gradient that had remained hidden in previous works due to technical restrictions. For this reason, some of the results in Cardoso 2017 came as a surprise to researchers that followed or were part of these early endeavors.

We do agree with the reviewer strategy to compare our results from Cardoso 2017 with the early work performed by many labs that defined how the Dorsal gradient is established in the Drosophila embryo. We are aware of all the allelic combinations studied then, and their effects on the embryonic cuticle, the nuclear Dorsal gradient, and Dl-target genes. However, we must point out that, by performing a quantitative analysis of the Dorsal gradient, we observed patterns that had not been detected. We clearly observed a decrease in ventral nuclear Dorsal in cact loss-of-function, as revealed in the graphs in Figure 1, Suppl Fig 1 and Suppl Fig 2 of Cardoso et al, where we thoroughly analyzed several cact allelic combinations. The differences in ventral levels of nuclear Dl that we showed could not be perceived in the early works of Roth 1989, 1991, Belvin, 1995 or Bergman 1996, since Dl gradients were revealed with immuno-histochemical methods, as compared to quantitative fluorescent antibody detection and in situ hybridization. Therefore, we, and the reviewers of Cardoso 2017, are convinced of the effects of cact loss-of-function in decreasing ventral nuclear Dl levels and consequent decrease in the size of the domain of ventral genes such as snail and twist.

Their crucial simulation of the cactus null condition (Figure 4 E) leads to a uniform nuclear Dorsal distribution (corresponding to dorsolateral DV positional values!). However, this cannot be the case. They cite the paper by Bergmann et al., 1996 in which independent germ line clone experiments with three amorphic loss of function alleles of cactus were described. One of them is a defined genomic deletion of the entire cactus gene. In all three cases Bergmann et al observed nuclear Dorsal gradients, albeit with a decreased slope Thus, polarity was not lost.
Most importantly there was no indication of decreased ventral nuclear Dorsal concentrations compared to wildtype.

> The most important data in Bergmann et al., 1996 to support the referee's argument is shown in Fig. 8, the sole figure where the Dl gradient in a cact loss-of-function mutant is shown. The experiment that produced these results has two important characteristics that greatly differentiates from the experiments used in our paper:
1. They used immuno-histochemical methods
2. There was no quantitative analysis of the Dorsal gradient.

Since this may preclude detection of differences in high nDl amounts in ventral regions of the embryos, it makes no sense that Fig. 8 experiment (in Bergmann et al 1996) could be used to challenge fluorescent immunostaining assays combined with rigorous computational image analysis of Cardoso et al, 2017, as detailed below. All other data cited in the Bergman 1996 paper using germline clones for loss-of-function cact alleles is either not shown or is presented as embryonic cuticles, thus no further information on the Dorsal gradient is provided that could enable comparison with data shown in Cardoso 2017.

Bergman 1996 generates germline clones for three alleles:

- cact[255], a P-element insertion and likely hypomorph, they cite observing a V1 cuticle phenotype but no figure is shown;

- cact[III18] is Df(2L)III18, which deletes cact, fzy and chi genes, plus recoded fzy and chi. They show a shallow Dl gradient for these germline clones and cite a V1 cuticle phenotype;

- cact[D13], that has a premature stop codon, they cite a V1 cuticle phenotype but no figure is shown.

Based on cact[III18] germline clones they observe an expansion of nuclear Dl in dorsal positions of the embryo, in agreement with our own loss-of-function analysis, where Dl is expanded dorsally, as well as expression of the lateral target gene sog. On the other hand, the Dl gradient shown for the cact[III18] germline clone does not allow to evaluate the Dl gradient in ventral regions of the embryo, a limitation of the staining technique, as it does not allow the linear assessment and thus quantification of the signal. Besides that, our quantitative analysis using fluorescent detection shows that loss of cactus function in the strong loss-of-function allele cact[A2] over a deficiency for cact results in ventral loss of peak nuclear Dorsal levels.

>However, we agree with the reviewer that our results, as well as those in the literature, do not show complete loss of polarity in cactus mutants. For that reason, we are grateful to the reviewer in pointing out an inconsistency in our writing. Fig. 4E shows model simulations for different levels of Cact reduction, from 80 to 100% reduction. All those simulations agree with our experimental data (Cardoso 2017) except the limit situation where Cact is completely absent. This discordance clearly indicates that there are additional mechanisms that may still need to be included in the model. However, as with any model, we understand that our model is limited in the sense that it is not a perfect reproduction of the biological system.

We also realize that our writing may have generated this misunderstanding, therefore we have modified the sentence referring to Fig 4E where it now reads:

"....the model correctly predicts that loss of cactus function progressively leads to flattening of the Dorsal gradient, reflecting the pattern of embryos with expanded lateral territories"(Cardoso, 2017)

Furthermore, to make it clear to the reader, and based on the results of cact germline clones, as pointed out by the reviewer, we have added the sentence:

"On the other hand, the complete absence of Cact (0%) leading to apolar embryos has no support in the literature. Germline clones for loss-of-function cact alleles, which lead to the highest degree possible of cactus loss, still retain some polarity, suggesting that other processes are involved in gradient formation, such as Dorsal phosphorylation (Govind et al, 1993), not reflected in our model."

Early germline clones by pole cell transplantations using one of these amorphic alleles (a cactus point mutation with an early stop codon deleting all ankyrin repeats) led to embryos with slightly expanded twist domains (Roth et al. 1991).

*> the fact that cactus performs an inhibitory role, as evident in the experiment with the cact[D13] allele cited above by the reviewer, is clear in the literature and also in our own papers. This does not exclude, however, that cactus also displays an additional, positive role. This is extensively discussed in Cardoso et al, 2017 and also in Fontenele et al, 2013, and should take into account the **relative levels** of Cactus to Dorsal.*

Further fundamental mistakes of the current paper are the comparative simulations of pelle and Toll KD embryos. The experimentally observed null mutations of these genes lead to identical phenotypes with embryos lacking nuclear Dorsal to the same degree (Roth et al., 1989). It seems that the authors assume that Tollrm9 is a null allele, which in fact it is lateralized Toll hypomorph.

> Thanks to the reviewer's comments we realize that this section of the manuscript was unclear. While this section is not the central issue in our manuscript, we are at fault for not having stated our reasoning behind the simulations in Fig 7. We wanted to simulate two different conditions that impair Toll signals: the first condition blocks transduction of the Toll signal at the level of the kinase that leads to Cactus phosphorylation, ubiquitination and consequent degradation; the second condition impairs Toll activation that is required for mobilizing Toll-responsive signaling complexes. The first condition mimicked well a pelle loss-of-function mutant, which generates dorsalized embryos, with a phenotype similar to a Toll loss-of-function. It is important to note that dorsalizing Toll phenotypes either result from null alleles or from recessive alleles that display mutations in the intracellular domain, probably impairing signal transduction.

The second condition blocks Toll signals at the receptor level, by impairing Toll activation that is necessary to mobilize signaling complexes, prior to transduction of the Toll signal. Surprisingly, this condition resulted in a nDl

phenotype that recalls the phenotypes of lateralizing Toll alleles Tl[rm9] and Tl[rm10], which are alleles that carry nucleotide substitutions in the region encoding the Toll receptor extracellular domain (Schneider at al, 1991). Such mutations in the Leucine-rich domains probably impair the interaction between homodimers and decrease the mobilization of signaling complexes. Although we were unable to reproduce the Toll null phenotype by decreasing the level of "activated Toll", exploring the lateralized phenotype that resulted may give us insight into the most important parameters that vary in this condition.
To make this section clear, as well as the respective figure legends, we have modified the text accordingly (Figure 7 legend and first paragraph at page 19). We thank the reviewer for pointing this out.

In their simulation the null mutants of Cactus and that of Toll lead to almost identical phenotypes which both do not agree with experimental observations. The fact that their simulations lead to results agreeing with the wrong phenotypes, casts serious doubt on the entire approach.

> As discussed above, we are not exploring in Fig 7 the phenotype of a Toll null, but rather a Toll condition that leads to lateralized embryos, and that helps understand what are the fundamental parameter changes that lead to expansion of lateral gene expression territories, as seen in cact allelic combinations and in Tl[rm9].

All available evidence suggests that Dorsal protein (monomer or dimer) can be subject to Toll induced phosphorylation in absence of Cactus and that this process is required to achieve the highest nuclear Dorsal concentrations. This explains why cactA2; Toll double mutants are lateralized at a ventrolateral level, while cactA2; Tollrm9 is mesodermalized (uniform highest nuclear Dorsal) (Roth et al., 1989).

> Indeed, the phosphorylation of Dl protein has an important role in the Toll pathway and is an additional aspect that would be very interesting to include in further models. However, in this manuscript we have concentrated in modeling the effects of Cactus, particularly considering the novel effects we have observed from our quantitative analysis of several cact mutant combinations.

Reviewer #2: Barros et al used a modeling approach to characterize the kinetics of nuclear Dorsal (Dl) gradient in early Drosophila embryos, and showed that the Dorsal-Cactus complex formation independent of Toll-signaling pathway is a key step to be added in the model to fully reproduce experimental data on nuclear Dl expression. Typically, it is known that Cactus (Cact) binds to Dl and blocks Dl's entry into the nucleus. Upon activation of Toll signaling pathway, Cact is phosphorylated and degraded, allowing freed Dl to enter the nucleus. The authors had recently shown that Cactus actually has an additional role to promote Dl nuclear translocation, especially in the ventral region where Toll signal is high. Since the mechanism of Cact's dual role in nuclear Dl regulation is not known, they used a new reaction-diffusion based model to characterize Dl dynamics. Using the model, they were able to show that in addition to Toll-dependent Dl translocation, a fraction of Dl-Cact complexes work independently of Toll pathway, and unbinding of Cact from the complex would allow direct entry of Dl into nucleus. This Toll-independent Cact regulation was crucial to elucidate the dual role of Cact – prevent and promote translocation of Dl protein. In cact mutant embryos, reduced Cact level would form fewer Dl-Cact complex, and consequently reduced number of Dl molecules can be freed from the Dl-Cact complex by activated Toll pathway, resulting in lower Dl level in cact mutant embryos, especially in ventral region. In the lateral/dorsal region where Toll signal is low, however, reduced Cact would allow more free Dl to directly enter nucleus, increasing basal Dl level in cact mutant embryos (dominant Toll-independent Cact regulation). Their model agrees with experimental data, including WT and various dl and cact mutant conditions. The manuscript is written logically, and dl/+, cact/cact, and dl/cact mutant data were used appropriately to test dual role of Cact and to better understand underlying mechanism of Cact regulation.

Yet, there are some comments and questions I have on the manuscript that could improve this manuscript's quality and clarity.
1) In Fig 7C, and SF1, how significant are these changes in basal levels of nDl or Cactus? For example, is the difference between 0.002 vs 0.003 significant (the authors claim that in some figure panels)? Does the trend hold with respect to some changes in parameters, or is this very sensitive to a certain set of parameters only?

Unlike 7C, the variations in 7F are significant as DlC ranges from 0.004 to 0.032. However, in both cases these variations contribute to significant changes in the levels of nDl (Fig. 7A, D) and in the levels of DlCT (Fig. 7B, E). In regard to parameter sensitivity, we conducted a detailed study as explained below.

2) Regarding the first question, how robust is the parameters with respect to changes?

> To answer to this question and improve the manuscript in respect to parameter sensitivity, we included four new paragraphs starting at page 30, line 698. The histograms show that most of the best individuals found by the Genetic Algorithm were very similar in the parameter space, indicating considerable stability of the solution, even when using an epidemic strategy to prevent the population from being dominated by solutions within a local minimum

3) Based on which data did authors use lower Cactus concentration to simulate dl6/+ condition in the model? I understand lower Dl level, but I missed the evidence for lower Cactus level in dl mutant

> The reduction in Cactus concentration in the model as a consequence of reducing Dl levels is considered in the first reaction $cDl^0 \rightarrow cDl^0 + C_f$*. We introduced this reaction based on several papers that report the decrease in Cactus levels in response to a decrease in Dorsal levels, seen both in western blots and antibody staining in whole mount or transversely cut embryos (Kubota and Gay, 1995; Belvin, 1995; Bergman, et al 1996). Importantly, Kubota and Gay, 1995 suggested that Dorsal regulates either cact mRNA stability or translation. This paper was cited in the previous version of the manuscript, page 5, after " We take into account non-uniform activation of Toll as in current models, two Dl nuclear translocation mechanisms, translational regulation of Cact by Dl [29, 30]", 30 being Kubota and Gay 1995.*

4) In Fig 3D, why does nDl level lower in lateral/dorsal side as well (it wasn't so in dl6/+)? Is there any experimental data to support the model data?

> Our dl[6]/+ experimental results, where Dl levels are decreased to 85% of wild type levels, show no decrease in nDl levels dorsally. It is possible that this difference between experimental results and the simulation shown in Fig 3D reflects a more significant decrease in total Dorsal protein than that attained in dl[6]/+. Accordingly, quantification experiments using a different dl loss-of-function allele (dl[1]) (Lieberman et al 2009) have shown a reduction in nDl levels on the dorsal side.

5) In the author's previous paper (2017 Development), the authors observed lateral expansion of Dl in cact mutant embryos. But, I don't see such evidence in the new experimental data in Fig 4B. Any comment on that?

>In Fig 4B, and 4A we show the gradient of cact[A2]/cact[011] embryos, which result in a more subtle increase in lateral nuclear Dorsal levels, compared to cact[A2]/Df. These two genotypes are explored in Cardoso et al Figs 1C and Fig 1D, respectively. Importantly, both allelic combinations lead to an expansion of lateral territories, as shown in Cardoso 2017, Fig 1K and 1H. Since the deficiency (Df) used in cact[A2]/Df also deletes loci that are not included in our model we have chosen not to simulate this condition in the current manuscript.

6) It took a while to understand how DlCT and DlC explains dual role of Cact. It would be nice if the authors clarify it a bit more. Personally, I understood it only after reading Discussion section. Maybe use some of these phrases in Results, when they first talked about it?

> We have added a few sentences explaining this dual role of Cactus in the results section, as suggested by the reviewer (page 14, end of first paragraph, line 295 to 299).

Reviewer #3: The model by Barros et al. focuses on determining what the roles might be of novel aspects of Dl/Cact interactions. Namely, they focused on the Toll-independent regulation of Cact, the Dl-dependent translation of Cact, and the differentiation between "activated" Dl and "non-activated" Dl. The Araujo lab has been discovering some of these novel behaviors of Cact for several years, and this paper appears particularly motivated by the discoveries in Cardoso et al., 2017, in which Cact was shown to have a dual role for the Dl gradient.

It must be absolutely stressed that this model is an important contribution to the field, and I am looking forward to seeing it published. Since the pioneering modeling paper by Kanodia et al. (2009), the Dl/Cact modeling community has completely ignored the role of phosphorylation of Dl by Toll in the formation of the Dl gradient. The role that Dl plays in upregulating Cact protein levels has also been summarily ignored, even though, (1) on the face of it, it appears to be a very interesting regulatory mechanism, and (2) it was discovered a quarter century ago. Thus, this model is needed.

In addition, I must also stress that the authors' approach is very important to the field of developmental biology: quantitative experiments combined with hypothesis-testing through mathematical modeling. While at times, new quantitative results may seem to be at odds with classical experimental observations (colorimetric reactions, cuticle preps, etc.), the quantitative nature of the work gives important nuance to phenotypes that were not previously appreciated, and at times, may serve as a corrective for incorrectly-interpreted qualitative observations.

All of that being said, the work has several drawbacks that still need to be addressed, both major and minor. Most of the major concerns stem from the authors ignoring or not taking into account all interactions that have been discussed in the Dl/Cact pathway. This is not generally a problem, as it is not strictly necessary for every new model to always have at least the same interactions as previously-published models. Along those lines, it can be argued that they did not intend the model to capture these interactions, and instead focused on the roles that their own novel

contributions might play in shaping the Dl gradient. However, there are two issues with this: first, this work discusses aspects of the Dl gradient that are impacted by the interactions that were left out. And second, some of their conclusions, such as the values of diffusivity, are justified with faulty reasoning, and instead may simply stem from the authors not taking all data/published interactions into account. More details on all of this below, but the general ways to rectify this problem are (1) to directly discuss the pros and cons of their model and conclusions in regards to leaving out previously-published interactions, and (2) not to emphasize the conclusions that may stem from the fact that they left those interactions out. As such, most of my criticisms could be answered by adding crucial discussion elements and changing the emphasis of some parts of the text.

> *We would like to thank the reviewer for his thorough review and for openly stating the importance of our approach to the field of developmental biology and that he looks forward to the publishing of our manuscript. We have very carefully analyzed all the concerns brought up by the reviewer and have answered to all his points below, as well as made changes in the manuscript when appropriate. Particularly, we have added a new paragraph to the Discussion section that displays more clearly the different elements that were added in our model, which are based on experimental evidence, and how they compare to previously published models (Discussion section, line 495). We believe that our current version has gained in clarity upon revision. We also find that our discussion is now better integrated with recent developments in the field, pointing out essential but in most part complementary differences among the models proposed and how they may favor future understanding of the Dorsal/Cactus regulatory network.*

Before I proceed with detailed criticisms, I feel the need to state that as a reviewer, I am in a bit of an odd position, coming to the party during the second round of reviews. I will attempt to provide comments in light of both the first and second submissions of the paper, the first round of reviews, and the authors' responses.

Major concerns (in no particular order):

1) The authors assume only "active" nuclear Dl can regulate target genes. The authors should state evidence for this. If no evidence is available, and this is an assumption by the authors, this would imply this "direct-flow" nDl stands in the place of n[DlC]: figures such as Fig 1D are very similar to Fig 3F from O'Connell and Reeves (2015).

> *Based on the reviewers comment, we have searched the manuscript for references to "active" Dl. In the one place we have found this term it has been removed. However, throughout the manuscript we do refer several times to "active Toll". We would like to strengthen that Dl* refers to Dl that is phosphorylated in response to Toll pathway activation. Phosphorylated Dl increases nuclear import (Drier et al., 1999) and is important to attain peak nuclear Dl levels in ventral nuclei. With respect to target gene regulation, on the other hand, it is admissible that both Dl phosphorylated in response to Toll (nDl*) and "direct flow" nDl may regulate gene expression. Roth et al., 1991 have shown that double mutants for the Toll signal and for a cact null allele are lateralized. Therefore, the increased level of Dl that enters nuclei by direct flow, due to loss of Cact inhibition in these embryos, is able to induce the expression of lateral genes. This indicates that phosphorylation of Dl in response to Toll is not a sine qua non condition for Dl transcriptional activity. On the other hand, phosphorylation may increase transcriptional activity, as Drier et al., 2000 has shown that loss of specific phosphorylation sites affects DV patterning while maintaining the overall nDl gradient format (Drier et al., 2000). This is also in agreement with vertebrate data on phosphorylated NFkB family proteins. This information has been added as a new paragraph to the discussion section, where we discuss the effects of the two Dl types (Discussion, starting at line 524).*

> *The reviewer also draws attention to the similarity between Fig 2D and Fig 3F from O'Connell and Reeves, 2015, suggesting that nDl^0 in this manuscript may stand for the same entity as n[DlC] in that paper. However, if we consider that Dl that enters the nucleus by direct flow is capable of DNA binding, then it should not correspond to n[DlC], since the binding of Cact to a Dl dimer interferes with DNA binding. On the other hand, we do not refute the possibility that Cact may enter the nucleus and bind Dl, and that this may impact the Dl gradient, as previously very well explored in O'Connell and Reeves, 2015. A possibility to be addressed in the future is whether both nDlC and nDl^0 have a compounded effect on the nDl gradient. Tricky experimental data addressing nuclear localization of Cact and Dl complexes will be necessary to define which protein complexes, such as 2Dl, 2DlC and Cact unbound to Dl, enter the nucleus during these stages. We acknowledge this possibility in the new corrected version of our manuscript. (same paragraph as above, in Discussion, starting at line 524)*

Both hypotheses account for the apparent discrepancy between the experimentally-measured levels of nuclear Dl and the need for the Dl *activity* gradient to drop to near zero levels. Thus, the authors should discuss the pros and cons of favoring their interpretation of "deconvolving" the nuclear Dl gradient into two components vs. the interpretation of Dl/Cact complex acting as the contributor to the basal levels of the gradient. The discussion should include how the phenotypes attributed to nuclear Dl/Cact complex could be explained by their approach or by alternative mechanisms.

> We completely agree with the reviewer that both hypothesis require the need for Dl activity levels to drop to near zero levels in dorsal nuclei. We have added a whole paragraph dedicated to this issue in the discussion section (Discussion, starting at line 524). In our model, the amount of Dl^0 in the nucleus is subject to all processes that control DlC formation. However, as stated above in response to major concern 1), both processes (free Dl flow suggested by the current model and Dl nuclear export by binding to Cact in the nucleus as suggested by O'Connell and Reeves, 2015) could take place in the embryonic blastoderm. It is also important to state that in our model, deconvolving the nuclear Dl gradient into two components is important to enable the addition of novel elements that control cactus independent of Toll activation. A new paragraph added to the beginning of the Discussion section tries to display more clearly the novel elements of our model (Discussion, starting line 495).

2) One of the paper's main results, that their model can account for the dual-role of Cact, has possibly been previously explained by the shuttling mechanism. The authors should discuss the pros and cons of favoring their interpretation of Cact's dual role over shuttling, including how the phenotypes described by shuttling (Carrell et al., 2017 and Schloop et al., 2020) could be explained by alternative mechanisms.

> As one of our main goals, the model we propose is able to reproduce the overall pattern of nDl gradients resulting from cact- mutants. In addition, as it incorporates novel and experimentally proven elements to the Dl-Cact network, it will allow to test additional elements that are independent of Toll in the future. We are unaware that the shuttling mechanism has been tested in this context, which would actually be a very interesting analysis to perform. It is fundamental to state that both processes are not necessarily excludent, but may perform complementary functions For instance, our model suggests that the "availability" of DlC is key to the dual effect of Cact. This availability could also impact the movement of these complexes along the DV axis.
When analyzing our simulations in normalized graphs (please see below, new Supplemental Figure 7 and stated in results, line 216-222 and 237-244), as suggested by the reviewer, we observe that our model does not reproduce the widening of the gradient in dl/+ heterozygotes. Such pattern is, however, well fit by the model in Al Asafen et al., 2020, which takes into account shuttling, Toll saturation and nuclear DlC. A future development of our model could incorporate some or all of these elements, leading to a better fit of some nDl data. On the other hand, current models do not allow us to test the effect of novel elements that control Cact and that we have been working on for at least ten years. For instance, the Calpain A protease, which has a clear effect on the nDl gradient (Fontenele et al., 2013), especially in ventral and lateral nuclei, acts exclusively on free Cactus, an element not included in any models to date. This reasoning has been included in the Discussion section (starting line 624).

One result of their simulations was that D_{DlC} is very low (more on this below). If true, this rules out shuttling. Therefore, if the authors wish to make that claim, it would be helpful to describe why having a low D_{DlC} is an important conclusion to their model. Alternatively, this result could be a side effect of the fact that they did not account for all known aspects of Dl/Cact interactions, or did not account for all experimental data. That would be acceptable, but if that is the case, the authors may want to acknowledge that and to downplay their result that D_{DlC} is low.

> We reanalyzed our data based on reviewers' comments. Detailed answer for this concern is present in the responses to the major concerns 5 to 7.

3) The authors investigate how reducing the maternal dl contribution affects the Dl gradient. Their endeavor has multiple drawbacks. First, their simulations fail to account for the change in shape of the Dl gradient: from roughly bell-shaped to flat-topped. As shown by Carrell et al., 2017, Toll saturation is a crucial mechanism that re-shapes the Dl gradient when Dl levels are reduced. Carrell et al. showed that without Toll saturation, all prior models predicted the Dl gradient to be exactly the same shape, regardless of the initial conditions on Dl. When normalized, wildtype Dl gradients lined up exactly on top of heterozygous ones. The authors should also attempt this normalization procedure to see if all of their reduced-[Dl] gradients (Fig. 3B,D) also fall on top of each other, and comment on the results.

> We thank the reviewer for bringing up this point about the shape of the Dl gradient. We have analyzed the normalized curves and found that, as pointed out by the reviewer, the gradients do not reproduce the flat top observed in dl heterozygotes. Thus, we have modified the text in the results section (lines 216-223; lines 237-244) stating that our model specifically reduces the nDl maximum but only approximates the lateral part of the curve. We have also added the normalized curves to a new Supplemental figure (Fig S7), and further point out this difference in

the Discussion section, stating that future simulations should be tested that incorporate Toll saturation into our model.

Second, the authors should investigate how or why the dl[6]/+ simulations cannot match the experimental data It is actually possible that Toll saturation is implicit in their model equations, which is all the more reason to try to adequately fit the dl[6]/+ data.

> As stated above, our model does not include Toll receptor saturation. This information has been added to the text (third section of Discussion, line 625)

Third, the authors state that their simulations do in fact replicate the altered shape of the dl[6]/+ gradients (lines 206-209 of the tracked-changes document). This is clearly not the case, and this claim should be removed. Instead, the authors should acknowledge that their simulations do not match the observed shape change. The ability of the 1xdl Dl gradient to match the wildtype at 20% and 50% DV coordinate was explained in Al Asafen et al., 2020, and can be traced to a combination of Toll saturation, shuttling, and the presence of Dl/Cact complex in the nuclei.

> This statement has been corrected as requested by the reviewer, in the Results section (lines 216-222 and 237-244). We compare our simulations as to those performed by Al Asafen et al., 2020, where they obtain a better fit of the nDl gradient at lateral positions of the embryo and replicate the widening of the gradient.

4) In the authors' words, the novel contributions of their model can be described as "two Dl nuclear translocation mechanisms, translational regulation of Cact by Dl, and the two pathways leading to Cact regulation: the Toll-regulated and Toll-independent pathways." I could not find any analysis that attempted to determine which of these three processes is responsible for their most important results. On the face of it, I suspect that only the "two Dl nuclear translocation mechanisms" is important. In particular, the fact that only DlC, and not cDl0, can bind to Toll, is the main reason for their most important results. Thus, the dual-role for Cact can be traced back to Toll-dependent phosphorylation of Dl, which in their model is mediated by Cact.

> This is a critical issue that the reviewer brings up. We have modified our text at several places throughout the manuscript to try to point out more clearly the main elements that allow cact to exert its dual role (Discussion, lines 495-513; 551-554). We find that the dynamics of DlC formation is at the center of this dual function. This interpretation is based on the placement of DlC in our network, capable of providing both Dl^0 and DlCT for the two nuclear translocation routes. It is also based on the differences in DlC levels observed in our mutant simulations and the observation that by increasing DlC formation (increasing k5) the cact loss-of-function phenotype is recovered (Fig 7G). On the other hand, in the network we have built, this central role for DlC cannot be dissociated from the two routes for Dl nuclear translocation. In addition, through the reaction $cDl^0 \rightarrow C_f$, (kinetic constant k1) Dorsal controls the concentration of the species (Cf) that binds to it and forms DlC. Thus the reaction is key to all novel contributions to our model as well.

Reviewer 1 also made mention of this, describing one of the novel aspects of the model as "a Toll-dependent mechanism by which Cactus promotes Dorsal nuclear transport." The authors responded by saying, "We actually suggested the inverse, that a Toll Independent mechanism of Cactus regulation controls Dorsal nuclear import…In fact, we believe that we made clear in the manuscript that a Toll-independent mechanism is being described…" Unfortunately, the authors did not make that point clear. In fact, in the Discussion, the authors state, "In forming DlC, Cact plays a negative role to impair Dl from entering the nucleus, while by enabling new DlCT complexes it favors Dl nuclear translocation." Perhaps I am misunderstanding what they are trying to say in the rebuttal to Reviewer 1.

> We understand that the Toll-independent processes where not clearly stated in the previous version of the manuscript. We have added a new segment to the beginning of the discussion section (line 495) that tries to establish more clearly the distinction between toll-dependent and independent processes. We have also modified Fig 1 with the same purpose.

As far as I can tell, the Toll-independent mechanism amounts to a first-order degradation of Cact, which is present in all Dl/Cact models up until this point. The novel regulation of Cact degradation comes from the fact that there is Toll-dependent production of C_ub, which is a species that has not previously been modeled. Indeed, previous models all assumed that Toll's role in Cact regulation was to split apart the Dl/Cact complex, and free Cact was subsequently degraded by constitutive mechanisms (i.e., a high first order degradation rate), which are mechanistically

indistinguishable from the authors' Toll-independent pathway. Furthermore, the fact that only DlC can bind to Toll, which then promotes Dl nuclear translocation at a rate higher than "direct flow," suggests that two aspects of the Toll-dependent mechanism are responsible for the promotion role for Cact: modeling of nDl* and the fact that only DlC can bind Toll.

> We believe that there is some misunderstanding in our use of the term "Toll-independent". We use the term as first proposed for Drosophila and vertebrate Toll-independent mechanisms: Toll-independent Cactus regulation, initially termed Toll-independent Cactus degradation (Belvin et al., 1995; Reach et al., 1996, Liu et al., 1997), involves processing and degradation of Cactus protein that is not bound to Dorsal and that does not involve Cactus phosphorylation and ubiquitination in response to Toll activity (Toll-dependent degradation). It rather involves the processing of a different pool of "free" Cactus by modulatory proteases of the Calpain family (Fontenele et al., 2009; Fontenele et al., 2013; Isoda and Nusslein-Volhard, 1994). It has been shown that Toll-dependent degradation requires N-terminal Ser residues of the Cact molecule, while Toll-independent degradation requires the C-terminal domain. Thus, they are two parallel processes. Toll-dependent and Toll-independent routes are conserved in vertebrates (Chen et al., 1995; Shen et al., 2001; Shumway et al., 1999; Schaecher et al., 2004), requiring N-terminal and C-terminal domains of IkappaBalfa respectively, and degradation by vertebrate calpains as part of the Toll-independent pathway.

Free Cactus, that is, unbound to Dl, has been detected in the early embryo (Isoda and Nusslein-Volhard 1994). Free Cactus can be degraded independent of Toll activation, but only Dl-bound Cactus is degraded in response to Toll through the proteasome. In our model a free pool of Cactus that is not bound to Dl can be degraded or can form new DlC complexes. Complex formation does not require Toll activity, consistent with the presence of DlC complexes in dorsalized embryos (Isoda and Nusslein-Volhard 1994), and is herein described as a Toll-independent mechanism. Free Cactus has not been included in published models of Dl gradient formation as a separate pool, neither has the Toll-independent degradation of free Cact or its role to build new DlC complexes. Degradation of Cact through the proteasome following phosphorylation and ubiquitination is a Toll dependent process, while degradation of free Cact is Toll-independent. Therefore, in our model, the part of the Toll-independent mechanism that controls Cact levels differs from other models in the literature in two aspects: (i) The reaction $C_f \rightarrow 0$ (kinetic constant k2) indicates the degradation of Cact molecules that had never interacted with Toll (this is why we call it Toll-independent); (ii) The reaction $cDl^0 \rightarrow C_f$ also contributes to the control of Cact concentration (this reaction is obviously Toll-independent as well).

> That being said, the reviewer is correct in stating that "two aspects .... are responsible for the ....role for Cact: modeling of nDl and the fact that only DlC can bind Toll". However, these two aspects are herein described as parts of two independent pathways, which have not been modeled as such previously. As stated above, we have modified several terms throughout the manuscript to make this distinction more clear to the reader, and added a whole section at the beginning of the Discussion stating clearly the most important aspects of our model.*

In summary for this point, what is novel is *not* the Toll-independent pathway for Cact degradation. All models had this. What is novel is (1) the modeling of a Dl* species that has different properties from Dl0, (2) the fact that only DlC can bind Toll to create Dl*, and (3) the creation of a C_ub species. (Of these three, I am fairly certain the third point is not very important.) Therefore, I implore the authors first to analyze their model further to determine which of their novel aspects is important for their main results, and in particular which pathway (Toll-dependent or -independent) is responsible for the promoting role for Cact, so that their conclusions can be discussed without confusion. (This would be an important analysis to do even without the confusion.) And second, to devote less discussion to the Toll-independent pathway, as this is not the novel aspect (all previous models had this). Instead, the novel aspect is the modeling of Dl*, together with their description of Cact-mediated binding of DlC by Toll.

> Considering our statements in the previous paragraph, we must insist that the Toll-independent mechanisms included in our model are novel. The Toll independent mechanisms are:
- Free Cactus degradation/processing (k2), not included in previous models
- Cactus protein synthesis from maternal mRNA, stimulated by Dorsal protein (k1), not included in previous models
- Mobilization of free Cactus to bind non-phosphorylated Dl in the cytoplasm and build new 2DlC complexes (k5, k6). This reaction was not part of an independent pathway in previous models, but was included.

All models included the recruitment of DlC to signaling complexes (herein referred to as reaction k7), which is dependent on Toll activation, as well as downstream steps to Dl nuclear translocation. The flow of Dl in and out of the nucleus was previously included in O'Connell and Reeves and further publications of the group, but as part of a complex with Cact.

> To make these points clear we have modified the text in the Discussion section, and changed a few terms throughout the text as stated above.

5) As mentioned above, the data-fitting results in a low DlC diffusivity. The authors state that this is “due to interaction with the Toll receptor,” implying that binding of DlC to Toll slows down its movement. This is not true. In their model, the diffusivity (intercompartmental exchange) of DlC is a free parameter that can be varied independently from DlC’s interactions with Toll. The *effective* or *apparent* diffusivity of DlC (which would be observed if watching the spread of DlC, and which might be characterized as D_{DlC}/(1 + Keq), where Keq is the equilibrium constant of DlC-Toll binding) could be low due to Toll binding, but not D_{DlC} itself. As a counter example, D_{Dl} is not low, even though Dl is captured by the nuclei; however, an *effective* diffusivity of Dl could be low. It seems strange they would explain the low value of D_{DlC} that way while at the same time acknowledging this does not apply to cDl, saying “the intercompartmental diffusion term…is much larger than the kinetic constants k3, k5 and k11.” At any rate, if D_{Dl} can be determined independently of the other kinetic parameters that might otherwise slow down Dl, so can D_{DlC}. The authors should revise their explanation for why D_{DlC} is low.

*> We agree with the reviewer that in our model the diffusivity of DlC (intercompartmental exchange) is a free parameter. However, as with any experimental data what is displayed is the *effective* or, formally defined as, apparent diffusion. This is a limitation that every reaction-diffusion model must deal with: the model diffusion parameter is an intrinsic characteristic of the molecule, but the experimental data reflects the apparent diffusion. Having that been said, we agree with the reviewer that we should revise our analysis about the diffusion parameters we have obtained. In that sense, we have greatly modified the discussion section "The balance between reaction and diffusion of free and complexed Dorsal and Cactus", pointing out that our values for Dorsal and Cactus diffusivity are likely overestimated. On the other hand, we have analyzed in more detail the diffusivity for the DlC complex based on suggestions made by the reviewer (points below) and find that they are in the range estimated by others.*

6) Along those lines, it is very surprising that the diffusivities of cDl and Cact dominate over other processes. First, intercompartmental exchange is slow (Delotto et al., 2007). Second, Cact is known to have a high turnover rate, so a slow intercompartmental exchange should not dominate a high degradation rate. Third, nuclear import of Dl, which has a time scale of roughly 1 min (eyeballing FRAP data from Delotto et al., 2007) or roughly 2-5 min (boxplots by Carrell et al., 2017), would be unlikely to be dominated by a process that apparently has a time scale of roughly 10 minutes. Indeed, the paGFP experiments by Carrell et al., 2017 suggest that nuclear capture dominates, as paGFP spreads only 6-7 nuclear diameters over 90 min on the ventral side, but spreads to fill the entire view of the embryo in 90 min when activated on the dorsal side (where no nuclear capture occurs).

> The reviewer is correct in pointing out this inconsistency. As stated above, we have greatly modified the section in the discussion that deals with this point. We believe, and made it clear in the revised manuscript, that the overestimated diffusion coefficient found by our model is related to the fact that cDl^0, nDl^0, Cact and DlC display uniform distribution even though they interacts with species that show non-uniformly distribution like DlCT and nDl. In order to correct it, we are presenting an alternative explanation for the behavior. These uniform distributions could be related to the high Cactus turnover, which has been shown experimentally.*

7) Also about diffusivity: In dimensional units, either the diffusivity of Dl is itself too high (and not just in comparison to other processes), or the authors’ time unit is too large. They state that D_{Dl} = 91 inverse time units (D_{C} is even larger). To put this parameter back into dimensional units, one must multiply by the square of the half-circumference of the embryo (about 300 microns), then divide by T = 1, which is in an unspecified time unit. This gives a dimensional D_{Dl} of 8.91e6 um^2 per (time unit). While the authors do not specify what their time unit is, to bring a number of 9 million down to the expected order of 0.1-1 um^2/s, that would require their time unit to be about a year: 9 million um^2/yr roughly equals 0.3 um^2/s.

Of course, if the time unit were a year, nuclear import of Dl would be 1 inverse year (or 2e-6 inverse minutes). In which case, Dl would effectively never enter nuclei. (Thus, two criticisms are at play here: acknowledge the too-high value of D_{Dl} required by their model, and explicitly specify their time scale.)

Given the predictions of their model being at odds with “known wisdom” (as in the case of Cact turnover) and quantitative measurements (both from point #6), the too-high value of D_{Dl} (this point), as well as point #5, the authors need to revise their discussion subsection on diffusivity. In particular, the authors should acknowledge their model makes diffusivity predictions opposite of what would be expected from known data (as to which processes dominate), and discuss this in light of the fact that their model focus was only on their own important, novel contributions. They should also acknowledge that their model predicts a quantitatively too-high value of diffusivity. All of these are potentially fine, as long as the limitations are acknowledged and not presented as strengths or major

predictions of the model. Not everything about every model needs to match with every expectation or previous experimental observation, especially if the model in question is leaving out previously-published interactions.

> Following the results published in Carrell et al., 2017, in particular considering the time scale of nuclear import of Dl of roughly 2-5 min, we have determined the typical time scale of our model, and then we have estimated the diffusion coefficients for Dl, Cact and Dl-Cact.

> We agree that our model overestimates the diffusion coefficient for Dl and Cact separately, as pointed by the referee. We made it clear in the manuscript and presented possible explanation for that, as described above. The DlC complex, on the other hand, has its diffusive behavior compatible with the system's time scale. We agree that it is a limitation of our model, and it is quite possible that the future insertion of phenomena such as Toll receptor saturation and shuttling may provide us with more realistic diffusion coefficients for the two species in question. We have made these changes as the reviewer suggested in the light of what our model was able to reproduce, and the challenges that it still needs to deal with when being improved.

Minor concerns (in no particular order):

1) The authors present their model as a series of PDEs, which they then discretize to match the compartments. This is incorrect. Their model was never a series of PDEs, because they always intended to discretize space into 50 compartments to match the different cytoplasmic regions of the syncytial blastoderm. If the model really were PDE-based at its foundational level, then the model results should not depend on how space is discretized. They should be able to discretize space into 40 or 75 compartments and get the same results. Given the geometry of the embryo, the model is actually, at its foundational level, a series of ODEs, with mass transfer terms that resemble, but do not originate from, a discretized second derivative. This should be fixed.

> Equations 2-10 presented in Supplementary Text are, in fact, Partial Differential Equations (PDEs), since we are modeling the concentrations of each species in the system as continuous variables in both space and time, i.e. C = C(x,t). Such equations are the starting point for solving our problem, and then we apply spatial discretization through a central second-order approximation (a finite difference method to discretize the second derivative operator over x), obtaining a system of Ordinary Differential Equations (ODEs) for each compartment, as described in the Methods section ("Model calibration and simulation"). If we do not take into account the geometric details of the Drosophila embryo, the problem described is independent of the number of compartments. In fact, if we choose a number of discretized regions greater than the number of points in the experimental data (these, in turn, corresponding to the number of cytoplasmic regions of the syncytial blastoderm), we would need to make an interpolation of the experimental data in order to solve Equation 11 of the Supplementary Text. That said, we agree with the reviewer that, when we consider geometric aspects of this biological system, the number of compartments must be equal to the number of cytoplasmic regions of the syncytial blastoderm to make our model suitable. Otherwise, we would need to handle issues such as the location and the size of the nucleus in each cytoplasmic region, and our model should be adapted to solve these factors. Therefore, we agree that, strictly speaking, our model should be better described as a sequence of ODEs with terms that connect adjacent compartments (the mass transfer element described by the reviewer). We have done these changes in our text, more specifically in the Methods section and in the Supplementary Text.

2) The authors should report their values of D_{Dl}/L^2 and D_{Cact}/L^2 in scientific notation, potentially with periods meaning decimal points rather than separations among groups of three digits. The typical reader would wonder what "1.037.500" means. As it was, until I did the calculation (91*50^2), I thought their value of D_{Dl}/L^2 was two hundred twenty seven point five, instead of 2.27e5.

> Considering the discussions from Major Concerns 5-7, we chose to report diffusion of Dorsal and Cactus in a different way, following the reviews made by the referee. Because of this, the analysis of D_ {Dl} / L² and D_ {Cact} / L² are no longer reported, giving rise to a discussion about time scales and the comparison of diffusion values in um²/s.

3) First sentence of the Results section, the authors claim that there are two different mechanisms for Dl nuclear transport. They further claim that cDl* is actively imported. I am not aware of a study that shows the *mechanisms* by which cDl0 and cDl* enter the nuclei are different, or that cDl* import is active. The paper they cite at the end of that sentence, Delotto et al., 2007, does not present any evidence that the mechanisms are different or that there is active transport. Indeed, in the very next sentence, the authors acknowledge there is no discrimination in mechanism in that paper, or any other paper for that matter. The authors should revise the first sentence to reflect this. Perhaps state that it has been shown that Dl* has a higher affinity for the nucleus (and cite the appropriate paper), which they take to mean the mechanisms of import are different, although there could be other interpretations (such as differing mechanisms of export).

> We completely agree with and thank the reviewer for this observation. We have revised the first sentence of Results, which now stands as " Embryonically translated from maternal mRNAs deposited in the egg, Dl protein nuclear translocation is regulated at two levels (Roth et al., 1991, Govind et al., 1993, DeLotto et al., 2007): high levels of nuclear translocation are dependent on Toll signals, that induce the phosphorylation and degradation of the Cact inhibitor, as well as Dl phosphorylation (Drier et al., 1999); basal levels of Dl shuttle in and out of all nuclei, as observed even in dorsal nuclei (DeLotto et al., 2007)."

Further mentions to different Dl "transport mechanisms" have been substituted by distinct Dl "entry routes".

4) Line 149 of revised submission with changes tracked: This is the only place that Fig. 2C is referenced in the manuscript. Fig. 2C has a depiction of gene expression domains, but nothing about gene expression is mentioned in the main text. The authors should elaborate.

> We have elaborated on the gene expression pattern line 158 (results section 1) and referenced the figure in the Results sections line 379.

5) Table 2: Why does the value of [Dl]tot change between dl[6]/+ and dl[6]/cact[A2]? Forgive me if this was answered elsewhere…I cannot find it.

> Indeed, the DlT values are slightly smaller for dl[6]/cact[A2] than dl[6]/+. It should be noted that so are the experimental nDl ventral peak and nDl simulated in our model. It is possible that these differences in total Dorsal fall in the range not detectable experimentally with usual techniques such as western blots. Alternatively, parameters absent in our model may account for this difference.*

6) Table 2, still: Can the authors elaborate on where the parameters [Dl]tot and [C]tot actually enter the model? Does [Dl]tot affect the initial conditions for Dl-containing species? What does [C]tot do? I could not find this information, so please forgive me if it was accessible.

> In the Supplementary Text, we report that "Total Dl concentration was a calibration parameter assumed to be uniform along the embryo (...)", and in the non-revised version, we mention that "we simulated only the 14th mitotic cycle, setting a uniform distribution of cytoplasmic free Dorsal cDl^0 as the initial condition". However, we agree that such information is not clearly stated in the Methods section itself. Therefore, we add an explanation, elucidating that, at the beginning of our simulation, we only have cDl^0, whose concentration is given by the total parameter [Dl]_{tot} in each compartment, and the activated Toll (whose profile is given by Equation 1 of the Supplementary Text). We thank the reviewer for giving us such a touch. As for Cactus, its concentration is not a parameter of the model, but a result obtained numerically. To calculate it after the fitting, we make a sum of all the species that contains Cactus (C_f, DlC, DlCT and C_ub), and a numerical integration afterwards. It is worth mentioning that the relationships involving total Dorsal and Cactus are described in Figure 1C ("Relationship among different species").

7) Lines 288-290, tracked-changes file: The authors state that nDl0 and nDl* have different DNA binding efficiencies. Where are they getting this information? There is no citation. This is essentially the same question as my first "Major" criticism, but pointed out in a different place in the manuscript where a citation is needed.

> This is a very important piece of information. We thank the reviewer for pointing this out. We have added this information in the discussion section together with appropriate references in the paragraph starting at line 524-532.

> We have also answered this question in more completeness above, as response to the first major criticism.

8) Lines 163-164: What is the experimental nuclear Dl concentration in the ventral-most region? Do the authors have a number for that, and a citation?

>Dl levels are defined experimentally by fluorescence levels and are non-dimensional.

9) The methods are lacking information about fly lines and experimental protocols. The extent of the Dl gradient in space appears to be closer to what was reported by Chung et al., 2011 and Kanodia et al., 2011, when the imaging was done only 70 microns from the pole. This has been clearly shown to result in a very extended Dl gradient. Is this how the embryo images in this paper were taken? What methods were used to fix and section the embryos? What antibodies were used? Also, the full genotypes of fly lines should be reported.

> *We have added this information to the methods section. Briefly, embryos were mounted in a microfluidic device and imaged at 90 μm from the anterior pole using a Nikon A1 confocal microscope. All genotypes and experimental procedures for the collection of quantitative data are included.*

10) The Gaussian shape for the activated Toll gradient is in a bit of an unusual form. Typically, there is a “2” multiplying the sigma-squared term in the argument’s denominator. This may seem nitpicky, but leaving that out makes it more difficult to compare the width of their Toll domain to parameters used in other papers. An unobservant reader may not realize they are not equivalent parameters.

> *The suggested change was made, and the Toll sigma parameter was adapted to take into account the factor of 2 mentioned by the reviewer.*

11) Fig. 6B: the authors characterize the Dl gradient’s ability to place boundaries by the slope of the gradient. In fact, the proper parameter is the slope divided by the concentration (1/c * dc/dx). Please revise accordingly.

> *The change was also made, and Figure 6B is in accordance with the reviewer's instructions.*

Have all data underlying the figures and results presented in the manuscript been provided?
Large-scale datasets should be made available via a public repository as described in the *PLOS Computational Biology* data availability policy, and numerical data that underlies graphs or summary statistics should be provided in spreadsheet form as supporting information.
Reviewer 3: No: Data associated with images and the images that went into creation of graphs are not available. Nor is the code that went into their mathematical model.

> *The experimental data were already published (Cardoso MA et al. A novel function for the IkappaB inhibitor Cactus in promoting Dorsal nuclear localization and activity in the Drosophila embryo. Development. 2017;144(16):2907-13.). We can provide the mathematical code if requested.*

PLOS authors have the option to publish the peer review history of their article (what does this mean?). If published, this will include your full peer review and any attached files.

If you choose “no”, your identity will remain anonymous but your review may still be made public.

Do you want your identity to be public for this peer review? For information about this choice, including consent withdrawal, please see our Privacy Policy.

Reviewer #3: Yes: Greg Reeves

References

1. Drier EA, Huang LH, Steward R. Nuclear import of the Drosophila Rel protein Dorsal is regulated by phosphorylation. Genes Dev. 1999;13(5):556-68.
2. Roth S, Hiromi Y, Godt D, Nusslein-Volhard C. cactus, a maternal gene required for proper formation of the dorsoventral morphogen gradient in Drosophila embryos. Development. 1991;112(2):371-88.

3. Drier EA, Govind S, Steward R. Cactus-independent regulation of Dorsal nuclear import by the ventral signal. Curr Biol. 2000;10(1):23-6.
4. O'Connell MD, Reeves GT. The presence of nuclear cactus in the early Drosophila embryo may extend the dynamic range of the dorsal gradient. PLoS Comput Biol. 2015;11(4):e1004159.
5. Al Asafen H, Bandodkar PU, Carrell-Noel S, Schloop AE, Friedman J, Reeves GT. Robustness of the Dorsal morphogen gradient with respect to morphogen dosage. PLoS Comput Biol. 2020;16(4):e1007750.
6. Fontenele M, Lim B, Oliveira D, Buffolo M, Perlman DH, Schupbach T, et al. Calpain A modulates Toll responses by limited Cactus/IkappaB proteolysis. Mol Biol Cell. 2013;24(18):2966-80.
7. Belvin MP, Jin Y, Anderson KV. Cactus protein degradation mediates Drosophila dorsal-ventral signaling. Genes Dev. 1995;9(7):783-93.
8. Reach M, Galindo RL, Towb P, Allen JL, Karin M, Wasserman SA. A gradient of cactus protein degradation establishes dorsoventral polarity in the Drosophila embryo. Dev Biol. 1996;180(1):353-64.
9. Liu ZP, Galindo RL, Wasserman SA. A role for CKII phosphorylation of the cactus PEST domain in dorsoventral patterning of the Drosophila embryo. Genes Dev. 1997;11(24):3413-22.
10. Fontenele M, Carneiro K, Agrellos R, Oliveira D, Oliveira-Silva A, Vieira V, et al. The Ca2+-dependent protease Calpain A regulates Cactus/I kappaB levels during Drosophila development in response to maternal Dpp signals. Mech Dev. 2009;126(8-9):737-51.
11. Isoda K, Nusslein-Volhard C. Disulfide cross-linking in crude embryonic lysates reveals three complexes of the Drosophila morphogen dorsal and its inhibitor cactus. Proc Natl Acad Sci U S A. 1994;91(12):5350-4.
12. Chen Z, Hagler J, Palombella VJ, Melandri F, Scherer D, Ballard D, et al. Signal-induced site-specific phosphorylation targets I kappa B alpha to the ubiquitin-proteasome pathway. Genes Dev. 1995;9(13):1586-97.
13. Shen J, Channavajhala P, Seldin DC, Sonenshein GE. Phosphorylation by the protein kinase CK2 promotes calpain-mediated degradation of IkappaBalpha. J Immunol. 2001;167(9):4919-25.
14. Shumway SD, Maki M, Miyamoto S. The PEST domain of IkappaBalpha is necessary and sufficient for in vitro degradation by mu-calpain. J Biol Chem. 1999;274(43):30874-81.
15. Schaecher K, Goust JM, Banik NL. The effects of calpain inhibition on IkB alpha degradation after activation of PBMCs: identification of the calpain cleavage sites. Neurochem Res. 2004;29(7):1443-51.
16. Carrell SN, O'Connell MD, Jacobsen T, Pomeroy AE, Hayes SM, Reeves GT. A facilitated diffusion mechanism establishes the Drosophila Dorsal gradient. Development. 2017;144(23):4450-61.
17. Govind S, Brennan L, Steward R. Homeostatic balance between dorsal and cactus proteins in the Drosophila embryo. Development. 1993;117(1):135-48.
18. DeLotto R, DeLotto Y, Steward R, Lippincott-Schwartz J. Nucleocytoplasmic shuttling mediates the dynamic maintenance of nuclear Dorsal levels during Drosophila embryogenesis. Development. 2007;134(23):4233-41.
