# Supplementary material for: A reaction-diffusion network model predicts a dual role of Cactus/IκB to regulate Dorsal/NFκB nuclear translocation in Drosophila
Source: PLoS Comput Biol. 2021 May 27;17(5):e1009040. doi: 10.1371/journal.pcbi.1009040 (PMC8189453; doi:10.1371/journal.pcbi.1009040)
Supplement: Attachment — Submitted filename: Answers to reviewers.pdf [file pcbi.1009040.s012.pdf]

Rio de Janeiro, April 22, 2021

Dear PLoS Computational Biology editors,

Please find below our responses to reviewer's comments concerning the manuscript entitled "A Reaction-Diffusion Network model predicts a dual role of Cactus/IκB to regulate Dorsal/NFκB nuclear translocation in *Drosophila*" by Barros et al. We would like to thank all the reviewers for helping us to best describe the strength and limitations of our results. This has truly been a very productive scientific discussion. We have modified the text as suggested, both in response to Reviewer 1 and in response to Reviewer 3, as described in detail below.

We are submitting a Revised manuscript (marked-up copy) showing all changes we have made to the last version of the manuscript. This file is being submitted as a "Related Manuscript" file, as required by the submission guidelines. All comments in our "response to reviewers" are made based on this version of the manuscript, with appropriate line numbering. Also, we are submitting a clean copy of our revised manuscript that does not show our changes. We have uploaded this as the "Manuscript" file, as required by the submission guidelines.

Cordially,

Francisco J. P. Lopes
Associate Professor
Universidade Federal do Rio de Janeiro - UFRJ
Campus Duque de Caxias
Developmental Biology and Dynamical Systems Group
flopes@ufrj.br

Answers to the reviewer's questions/comments

Reviewer's Responses to Questions
Comments to the Authors:

Reviewer #1: The authors produce a model for how Cactus is involved in Dorsal nuclear entry. Two routes are suggested. Route one requires activated Toll receptors and leads to phosphorylation and degradation of Cactus. Route two depends on direct reversible flow of free Dl dimers into the nucleus. The Dl dimers can associate with free Cactus, which itself is subject to Toll-independent processing. The authors first show that they can fine-tune the 18 parameters of their model so that their simulations reproduce the gradient of wt, dl heterozygosity, a hypomorphic cactus allele combination and cact/dl double heterozygotes. With model parameters fitting the data they try to simulate more a severe loss of cactus including the absence of cactus. This leads to a clear disagreement with published data and, according to the authors, suggests additional mechanisms not incorporated in the current model. However, this mechanisms uncovered by a null mutation are likely to contribute to the wt, dl heterozygous, cactus reduced and transheterozygous phenotypes they have simulated. Thus, the fact that their model fits to these data is a result of parameter fine-tuning and does not reflect the validity of the mechanistic assumptions.

>The proposed model is able to reproduce the wild type phenotype and three independent loss-of-function genotypes, two of which had not been simulated previously. Despite this concordance between data and simulations, there are some experimental data not reproduced by the model. However, as commented by referee 3 in the previous review, it is not expected that a model should be able to reproduce all known experimental results. If this were a current requirement, it would cause a long delay in the modeling of biological systems. Nonetheless, we have expanded the results section concerning the cact null (lines 338-348) to better discuss the possible reasons for this discrepancy.

The same applies to the authors attempt to simulate the loss of Toll activity. It makes perfect sense to take the reduction of DICT as a proxy for the loss of Toll activity. However, their simulation of the complete loss of Toll activity, i.e. DICT = 0 leads to lateralized phenotype, which again does not correspond to experimentally observed situation. I cannot see how the comparison to the special hypomorph Tlrm9 is helpful, here.

> We have revisited this section and analyzed in greater detail the $Tl^{act} = 0$ pattern observed. We agree with the reviewer that the comparison with the Tlrm9 lateralized phenotype was unhelpful to explain the apparent disagreement with published data and have thus entirely removed this section (results section lines 472-487). On the other hand, by re-examining the different species that vary as a result of this simulation, especially when compared to pll-, we notice that the nDl pattern displayed is misleading if one does not consider independently the pattern of nDl0 and nDl. At $Tl^{act} = 0$ cDl0 is still capable of entering the nucleus, contrary to pll-, where most cDl is immobilized as a DlCT complex. As a result, in pll- the levels of nDl0 are necessarily smaller than in $Tl^{act} = 0$. While both pll- and Tl- have been reported to generate a dorsalized phenotype, the amount of nDl in these mutant conditions has not been quantified. Furthermore, there are currently no methods available to determine whether nDl detected by immunolabelling in these Drosophila mutant embryos corresponds to nDl* or nDl0. Since at $Tl^{act} = 0$ no nDl* is expected, it is possible that the amount of nDl we observe in the Tl- simulation is solely nDl0, which is less efficient than nDl* in driving target gene expression. As a result, dorsal-lateral gene expression would ensue, closely related to the expected dorsalized Tl- phenotype.*

Taken together, the revision has not eliminated my main concerns about this study. The inability of the model to reproduce the phenotypes of cactus and Toll null alleles casts serious doubts about its basic assumptions.

>We have expanded the sections concerning the cact and Toll null phenotypes. As well pointed out by the editor, it was necessary to give a more thorough explanation as to why we believe our model is unable to reproduce the specific conditions of the cact and Toll null genotypes. Alterations were made in the Results section, lines 341-350 (for the cact null) and lines 460- 487 (for the absence of activated Toll).

Reviewer #3: In this submission of the manuscript, the authors addressed almost all of my previous criticisms. By adding more discussion, they presented the strengths of the model evenhandedly while still honestly acknowledging the small number of drawbacks of the model. However, there are still two criticisms that must be addressed. These criticisms are minor, in that they can be easily addressed by adding or deleting a few sentences, yet they are still important. Given they require such small edits, I trust the authors to be able to make the edits without needing to review the manuscript a third time.

I believe both of these criticisms stem from semantics, and after I present the details of the criticisms below, I offer a simple solution where the authors re-word a few things to clarify.

First and foremost, the authors continue to claim they have introduced a novel Toll-independent pathway for the degradation of Cactus. In my first review, I mentioned multiple times that all previous models had this. In their resubmission (the third, but only the second one that I reviewed), they added in multiple places (lines 30, 497, 500) the opposite statement: no other models had this. This is false: every other model had a term in the Cact equation that was the negative of a rate constant times the Cact concentration. Every other model assumed free Cact was degraded constitutively, and that Toll signaling simply split apart the Dorsal/Cactus complex to expose free Cact to the constitutive (Toll-independent) degradation.

The good news is that, as mentioned above, the authors introduce something else novel: the Toll-dependent pathway for the degradation of Cactus. No other model had Cact_ub as a species. So the novelty of their model, as far as Cact degradation is concerned, is the Toll-dependent ubiquitination of Cact, which allows for a different degradation rate than the Toll-independent degradation rate. So when the authors stated that no other model had the Toll-independent degradation of Cact, I think what they meant is that, since no other models had the Cact_ub species (which is the real novelty of the authors' model), then no other model had the ability *to distinguish between* the two pathways.

> We thank the reviewer for very interesting suggestions as to how to refer to the novelty of our model. Indeed, we agree that, in terms of the new reactions and terms that have been included in our model, they are part of the Toll dependent pathway that controls Cactus. This is true both for Cactus control by phosphorylation and degradation, as well as for the binding of free Cactus to Dl. Nonetheless, these additions are essential to distinguish between the Toll- dependent and Toll-independent pathways for Cactus control, as well observed by the reviewer. Therefore, we have modified our text in several places to correct how we state the novelty of our analysis. We have changed the abstract (lines 28-33), Introduction (lines 91-92, line 103, and lines 119-127), and the first section of the Discussion (lines 521-542 and 550-552).
We have also adjusted a few "Toll-independent" terms throughout the manuscript to make our statements clear. These modifications are all highlighted in the document with track changes.

Second, the same could be said for the semantics of free Cact binding to Dorsal. On line 503, they claim this is a novel term in their equations, but then admit it was previously found in other models while stating that previous models did not have it "as part of an independent pathway." However, the term in the model that describes this interaction is the exact same as the corresponding term in all other models: a rate constant times the concentration of free Cact times the concentration of free Dorsal.

Again, the good news is that this model does have something different that no other model before it had: an "activated" form of Dorsal, which cannot be bound by Cact. So again, as the first criticism, the novelty is

not in the Toll-independent pathway, but in the explicit modeling of the Toll-dependent effects (here modeling Dl*, and above modeling Cact_ub). Here this also allows the authors *to distinguish between* the two pathways.

> *As done for the previous point made by the reviewer, we have adjusted the text in order to correctly state the new terms added to our model and where lies its novelty in relation to the interactions between Cact and Dl. We have modified our text in several places: Introduction (lines 124-125), Discussion (lines 537-542)*

One more point about this second criticism: the authors state, as part of this novel term, that they have modeled "Mobilization of free Cactus." I cannot tell what they mean by "Mobilization;" it only appears one other place in the text (in a slightly different context). I suggest the authors remove this term.

> *We have substituted this term by "displacement"*

In general, both of these criticisms seem to be semantic: the authors intended the Toll-independent mechanisms to be the novel parts of the model, but what they have in fact done is add novel components of Toll-dependent action. This is proven by the fact that the Toll-independent terms in their model show up in the exact same way in all other Dorsal/Cact models. However, they have two novel species in their model: Cact_ub and Dl*, which no other previous model had, and which are both dependent on Toll.

Perhaps a middle ground could be forged, in that the authors could state that their goal was to determine the relative contributions of the two pathways, and in order to do that, they had to distinguish them by explicitly modeling the Toll-dependent species. That would highlight what is novel about their model (Cact_ub and Dl*) while also highlighting what they think is important: the difference between the Toll-dependent pathway and the Toll-independent pathway. As such, I recommend the authors do the following:

- Instead of saying their Toll-independent terms are novel, say the Toll-dependent terms (species) are novel

>*This has been corrected as suggested*

- State the motivation for explicitly modeling the Toll-dependent species is that they wanted to determine the relative contributions of the Toll-independent vs Toll-dependent pathways, and that in order to do that, they needed the novel Toll-dependent species.

>*This has been corrected as suggested*

- Keep any language that states the importance of the Toll-independent pathway. Previous models could make no such claim, because they didn't differentiate between the two pathways.

>*This has been corrected as suggested*

- To do this, they will have to edit lines 495 – 508 at the very least.

>*This has been corrected as suggested. In addition to changing these lines, as part of the discussion section, we have modified additional sections as stated above.*

I should note here that, even though these criticisms stem from semantics, if the authors do not correct how they present the impact of their paper, many readers would be confused about the state of the field and how their paper fits into it. So the criticisms must be addressed.

One minor issue that is not associated with the above disagreements: the sentence starting on line 770 of the manuscript with changes highlighted should be edited. I believe the authors forgot to delete “Partial Differential Equations System (PDEs).”

>*This has been corrected as suggested*

Other minor typos could be found throughout, so the authors should edit for grammar.

> *We have searched the document for typos and these have been corrected*
